# Assembly of Fillable Microrobotic Systems by Microfluidic Loading with Dip Sealing

**DOI:** 10.1002/adma.202207791

**Published:** 2023-02-26

**Authors:** Rujie Sun, Xin Song, Kun Zhou, Yuyang Zuo, Richard Wang, Omar Rifaie-Graham, David J. Peeler, Ruoxiao Xie, Yixuan Leng, Hongya Geng, Giulia Brachi, Yun Ma, Yutong Liu, Lorna Barron, Molly M. Stevens

**Affiliations:** Department of Materials, Imperial College London, London SW7 2AZ, UK; Department of Materials, Imperial College London, London SW7 2AZ, UK; Department of Materials, Imperial College London, London SW7 2AZ, UK; Department of Bioengineering, Imperial College London, London SW7 2AZ, UK; Department of Materials, Imperial College London, London SW7 2AZ, UK; Department of Materials, Imperial College London, London SW7 2AZ, UK; Department of Materials, Imperial College London, London SW7 2AZ, UK; Department of Materials, Imperial College London, London SW7 2AZ, UK; Department of Materials, Imperial College London, London SW7 2AZ, UK; Institute of Biopharmaceutical and Health Engineering, Tsinghua Shenzhen International Graduate School, Tsinghua University, Shenzhen 518055, China; Department of Materials, Imperial College London, London SW7 2AZ, UK; Department of Materials, Imperial College London, London SW7 2AZ, UK; Department of Metabolism, Digestion and Reproduction, Imperial College London, London SW7 2AZ, UK; Department of Materials, Imperial College London, London SW7 2AZ, UK; Department of Materials, Imperial College London, London SW7 2AZ, UK; Department of Bioengineering, Imperial College London, London SW7 2AZ, UK; Institute of Biomedical Engineering, Imperial College London, London SW7 2AZ, UK

**Keywords:** environmental sensing, fillable microrobotics, microfluidics, micromotors, targeted delivery

## Abstract

Microrobots can provide spatiotemporally well-controlled cargo delivery that can improve therapeutic efficiency compared to conventional drug delivery strategies. Robust microfabrication methods to expand the variety of materials or cargoes that can be incorporated into microrobots can greatly broaden the scope of their functions. However, current surface coating or direct blending techniques used for cargo loading result in inefficient loading and poor cargo protection during transportation, which leads to cargo waste, degradation and non-specific release. Herein, a versatile platform to fabricate fillable micro-robots using microfluidic loading and dip sealing (MLDS) is presented. MLDS enables the encapsulation of different types of cargoes within hollow micro-robots and protection of cargo integrity. The technique is supported by highresolution 3D printing with an integrated microfluidic loading system, which realizes a highly precise loading process and improves cargo loading capacity. A corresponding dip sealing strategy is developed to encase and protect the loaded cargo whilst maintaining the geometric and structural integrity of the loaded microrobots. This dip sealing technique is suitable for different materials, including thermal and light-responsive materials. The MLDS platform provides new opportunities for microrobotic systems in targeted drug delivery, environmental sensing, and chemically powered micromotor applications.

## 1 Introduction

Microrobotic systems have been widely explored in the last decade and have shown great potential in various biomedical applications, including diagnostics and therapeutics.^[1]^ Compared to traditionally passive nanomedicines, microrobots are active-matter systems composed of actuatable components including magnetic,^[2]^ acoustic,^[3]^ chemical,^[4]^ and/or materials of biological origin.^[5]^ These properties enable microrobots to navigate their environments and to perform highly specific tasks, such as penetrating deep tissues for drug delivery. Importantly, microrobotic systems can be engineered to perform different functions including targeted drug delivery,^[6]^ cell delivery,^[7]^ sensing,^[8]^ and imaging.^[9]^ However, the human body consists of complex microenvironments with varying pH levels, pressures, and size constraints. Designing microrobots with properties that permit navigation through complex environments like the body while maintaining efficacy is a multifaceted challenge.

Surface coatings are widely used as a direct approach for loading cargoes onto microrobots. These coatings capitalize on the interface forces between the micro-robotic devices and the loaded cargoes and can be released in response to exogenous or endogenous stimuli. For instance, hydrogen bonding has been employed to non-covalently attach surface-loaded drug cargoes that can then be released via external ultrasound stimulation^[10]^ or thermal perturbation.^[11]^ Microrobots can also be surface-modified to respond to changes in the chemical microenvironment (e.g., variations of pH) to trigger drug release.^[12]^ Apart from being drug carriers, micro-robots have also been employed as cell delivery and transplantation agents via surface attachment.^[13]^ However, surface coating can lead to undesired exposure of healthy tissues to potentially cytotoxic drugs upon administration and reduced dosage at the target site, which greatly limit their therapeutic efficiency.

Additive manufacturing with payloads of interest blended into a biodegradable precursor solution offers a more direct means of microrobot loading-by-synthesis. In particular, hydrogels have received significant attention in these applications due to their biodegradability and biocompatibility, which are crucial conditions for cargo release using this method.^[14]^ They have been employed as fully biodegradable cargo carriers with excellent cell biocompatibility^[15]^ and printability for microscale devices.^[16]^ Nevertheless, because direct cargo-blending is dependent on cargo–material interactions, new material formulations must be customized for new cargoes and are not easily translated to the delivery of biomacromolecules.

Despite the progress made, there are numerous unresolved challenges in functionalization of microrobots. First, it is difficult to achieve high cargo loading efficiencies using current methods, resulting in wastage during fabrication and inefficient delivery in vivo. Second, many of the current loading processes require chemical modification of the cargo to enable covalent attachment or adhesion to the microrobot.^[10]^ In addition to fundamentally altering the cargo, these methods also introduce fabrication limitations, including light sensitivity during 3D printing or low solubility when blending into printing resins.^[17]^ This is especially true for surface-coated microrobots as the cargo remains exposed within the body, leading to reduced cargo integrity and non-specific delivery profiles.^[18]^

In contrast to surface-coating and drug-blending techniques, capsule-like reservoirs can offer cargo protection and controlled release. Previous work has shown that cargoes encapsulated in hollow compartments can retain their physicochemical properties under a variety of conditions for on-demand release^[19]^ or controlled polymerization.^[20]^ Furthermore, cargo encapsulation has also facilitated pulsatile biomacromolecule release from hollow microparticles, which could be tuned by altering poly(lactic-*co*-glycolic acid) degradation rate and microparticle geometry.^[21]^

**Scheme 1:**
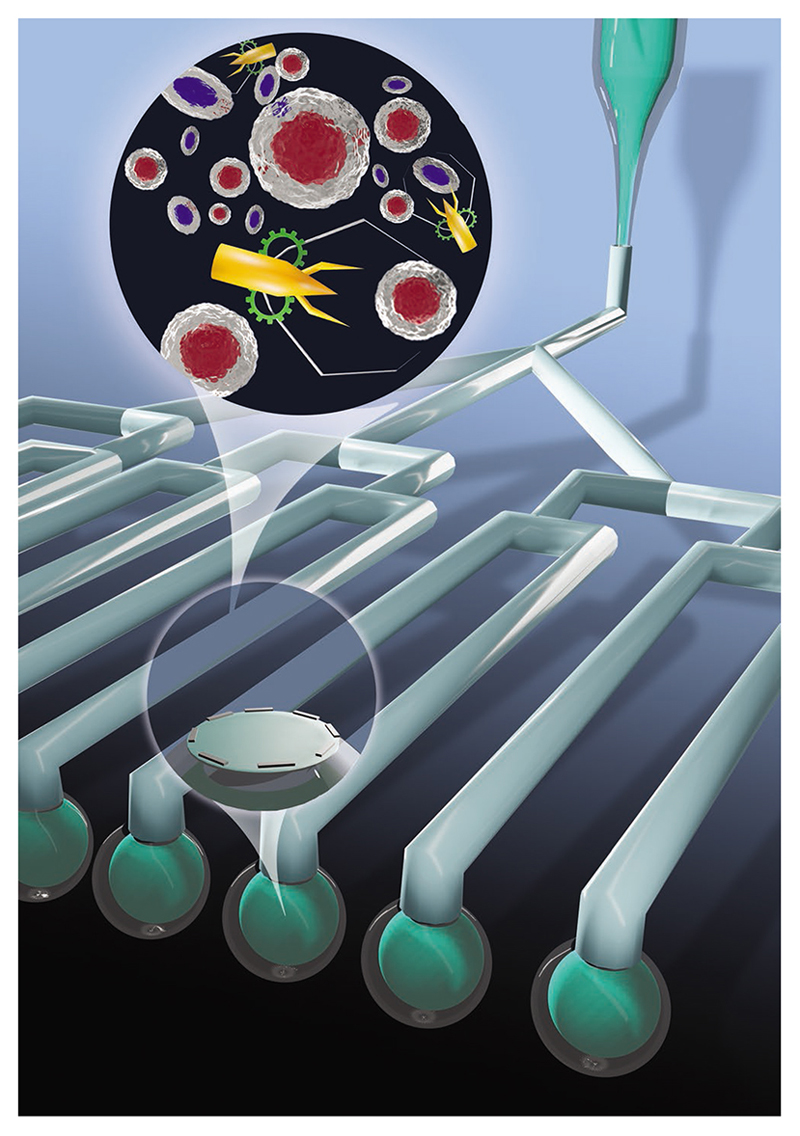
Schematic of fillable microrobotic system assembled by MLDS.

In this work, we present an integrated technique to fabricate fillable microrobotic systems via microfluidic perfusion and precise sealing, termed microfluidic loading with dip sealing (MLDS) (Scheme 1 and Movie S1, Supporting Information). We then describe how this technique was used to load diverse cargoes into hollow microrobots for various applications. In brief, the MLDS system was fabricated using two-photon polymerization (2PP)-based 3D printing. This platform was used to precisely load cargoes into the reservoirs of the microrobots with controllable liquid volumes, thereby increasing loading efficiency and minimizing wastage. Then, we developed a precise-contact dip sealing method to enclose and protect the loaded cargo within the microrobot reservoir with a stimuli-respon-sive sealing layer whilst preserving the microfeatures of the microrobot.

## 2 Results and Discussion

### 2.1 Design and Fabrication of the MLDS System

**Figure 1:**
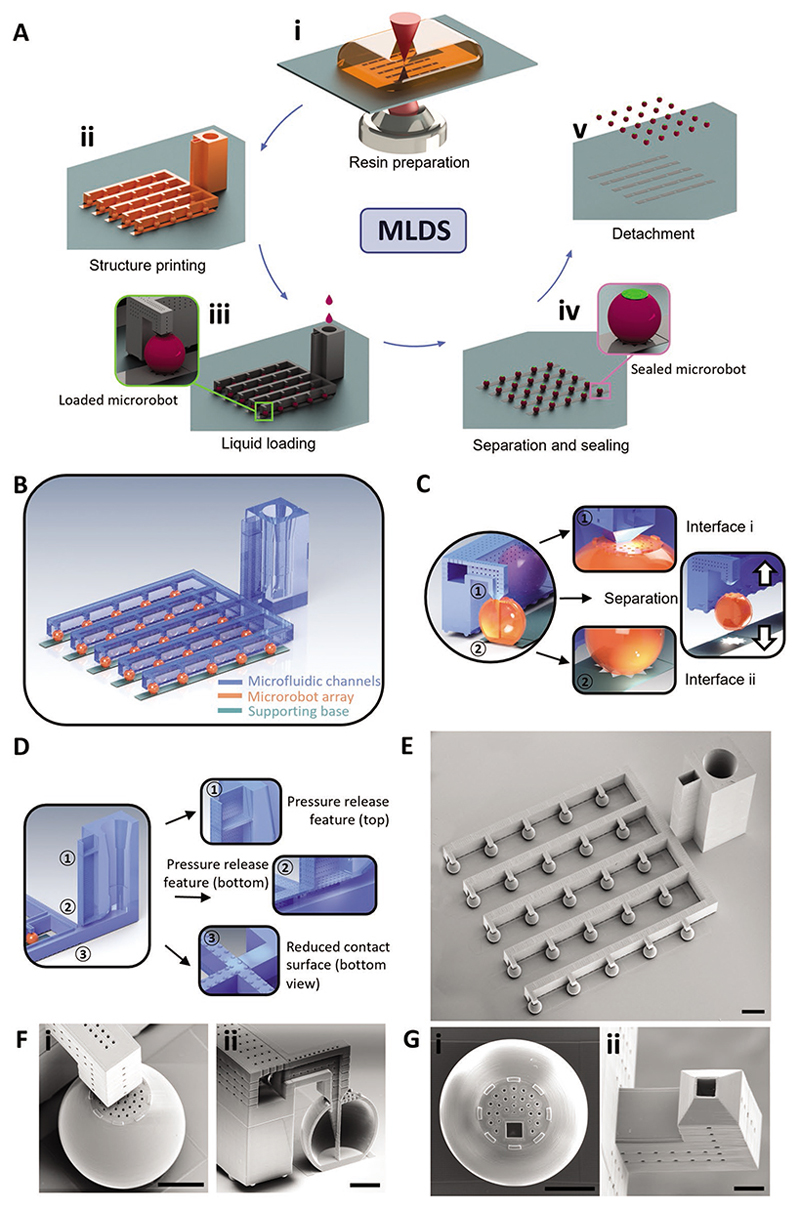
Design and fabrication of the fillable microrobotic platform by MLDS. A) The fabrication process for the MLDS system. i) IP-S is drop-cast onto a clean glass substrate. ii) The integrated system is printed and cleaned. iii) Cargo is loaded into the microrobot array by the microfluidic loading process. iv) The loaded microrobot array is sealed by the dip sealing method. v) The sealed microrobots are detached from the substrate. B) Schematics of the micro-fluidic loading system with three main parts: a supporting base, a fillable microrobot array, and microfluidic channels. C) Design concept of two interfaces between three main parts. Interface (i) is a weakened breakaway feature between microfluidic channels and microrobot inlets. Interface (ii) enables easy separation and harvesting of loaded microrobots from the supporting base. D) Design concept of the microfluidic channels. The pressure release feature is a reservoir with two porous layers, labelled in the first and second positions. The bottom of the microfluidic channels has reduced contact with the glass substrate for easy separation. E) SEM image of the overview of the printed microfluidic loading system. Scale bar: 500 μm. F) i) SEM image around the interface between the microfluidic channel and microrobot. ii) SEM image of the section view of the microrobot with the guiding channel extended into the microrobot chamber. Scale bar: 100 μm. G) SEM image of the microrobot (i) and microfluidic channel (ii) after separation. Scale bar: 100 μm.

The MLDS system is composed of two subsystems for loading and sealing, respectively. 2PP-based 3D printing was employed to fabricate the integrated microfluidic loading subsystem. The technique employs non-linear two-photon absorption to selectively polymerize the substrate resin within a rapidly scanned confocal voxel,^[22]^ which provides the printing resolution that is required to create the micron scale features of the microrobots in the MLDS system. The fabrication process of the MLDS system was divided into five steps (Figure 1A): i) the liquid resin used for printing was first drop-casted onto a clean glass substrate, covering the printing area. To improve adhesion between the printed structure and the glass substrate, the glass substrate was rendered hydrophilic via oxygen plasma treatment and chemically silanized prior to drop casting; ii) printing parameters such as laser power, scan speed, and shell printing algorithms were optimized to minimize printing time whilst maintaining structural integrity. Following print completion, the excess uncured resin was removed by sequential immersion of the substrate in propylene glycol monomethyl ether acetate (PGMEA) and isopropanol (IPA). As the microfluidic channels required longer cleaning process times, a temperature increase to 45 °C in PGMEA was used to accelerate the process whilst preserving the structure through a plasticization process. To improve the cleaning process, the microfluidic channels also featured a pore array distributed along the microfluidic channels that provided resin escape pathways. Ultraviolet (UV) post-curing was then employed to increase the overall stability of the printed structure by curing the residual non-polymerized resin: iii) the UV-treated microrobots were then loaded via rate-controlled syringe pump infusion of the cargo through the microfluidic array. One important advantage of the MLDS is the precise and direct route that cargoes take when loaded into microrobots, which improved loading efficiency and reduced stock volumes needed to achieve adequate loading. Importantly, unused cargo in the microfluidic channels could be recycled, thereby conserving expensive cargoes and reducing resource wastage; iv) the filled microrobots were separated from the microfluidic channels and encapsulated for the protection of the loaded cargo using a dip sealing process; and v) finally, the fully loaded and sealed microrobot array was detached from the glass substrate through mechanical perturbations.

To develop MLDS, we first designed an integrated microfluidic subsystem composed of three parts: a supporting base, a microrobot array, and microfluidic channels (Figure 1B). These three subsystems were 3D printed onto glass substrates via 2PP (Figure S1, Supporting Information) using the material of IP-S photoresin (Nanoscribe) which is a carbamate/methacrylate-based compound yielding polymers with high mechanical strength and stability.^[23]^ The collective system was composed of a microrobot array connected via microfluidic channels. Each microrobot had a diameter of 310 μm, a wall thickness of 15 μm, and a square inlet with a 35 μm edge (Figure S2, Supporting Information). A physical connection between the microfluidic channels and the microrobots was essential for loading cargo into the microrobot reservoir by continuous fluid flow. To facilitate microrobot separation after loading, we designed sequential breakaway interfaces between the subsystems (Figure 1C). The first breakaway interface was designed to separate the micro-fluidic channels from the microrobots after loading, followed by the second breakaway interface separating the microrobots from the supporting base after sealing. We envisioned that this design would ensure that the loaded microrobots remained on the glass substrate when separated from the channels and before being sealed. Both sequential separations were manually initiated through mechanical perturbations (Figure S3, Supporting Information). The first interface between the microfluidic loading channels and the microrobot was designed to connect the square inlet and to extend into the microrobot body with a guiding channel (Figure 1C, Interface i). This inlet served to improve the overall loading efficiency by reducing the effects of surface tension, which would otherwise prevent cargo from entering the microrobot. Cargo loading was performed by automated infusion through the microfluidic channels into the microrobot array. We envisioned that a build-up in pressure would be generated as the fluidic flow could not be interrupted immediately once all microrobots were fully loaded, which would cause a leakage that would contaminate the system. To reduce the risk of such leakage, we included a pressure-release feature with a two-layered reservoir. The bottom layer of this reservoir consisted of an array of intermediate size pores connecting the fluidic loading channels, and the upper layer consisted of an array of smaller pores exposed to atmospheric pressure (Figure 1D). The larger pores of the bottom layer acted as a fluid escape path to help relieve pressure, whilst the smaller pores of the upper layer provided sufficiently high surface tension to prevent liquid escape. A reduced contact design was included between the bottom of microfluidic channels and the glass substrate so that the microfluidic channels could be fully detached from the substrate after the microrobots were loaded. The design features were clearly confirmed by scanning electron microscopy (SEM) and optical images of the integrated microfluidic platform (Figure 1E and Figure S4, Supporting Information).

The breakaway interface between the microrobots and the microfluidic channels ensured both continuous fluid flow during cargo loading (Figure 1F) and subsequent separation of loaded microrobots from the channels. The small pore array on the lid was implemented to all the air inside the microrobot chamber to escape during fluidic cargo loading. SEM imaging of the microrobots after separation from microfluidic channels showcased the robustness of the design in retaining structural integrity and intact features (Figure 1G–i and Figure S5A, Supporting Information). This was particularly important as a clean surface was required to seal the microrobots after cargo loading. SEM imaging also confirmed that the microfluidic channel array was completely separated from the microrobots with the outlets remaining unbroken (Figure 1G–ii and Figure S5B, Supporting Information). Importantly, we successfully fabricated microrobots that could be fully filled with liquid cargo.

### 2.2 MLDS Working Process and Characterization

**Figure 2:**
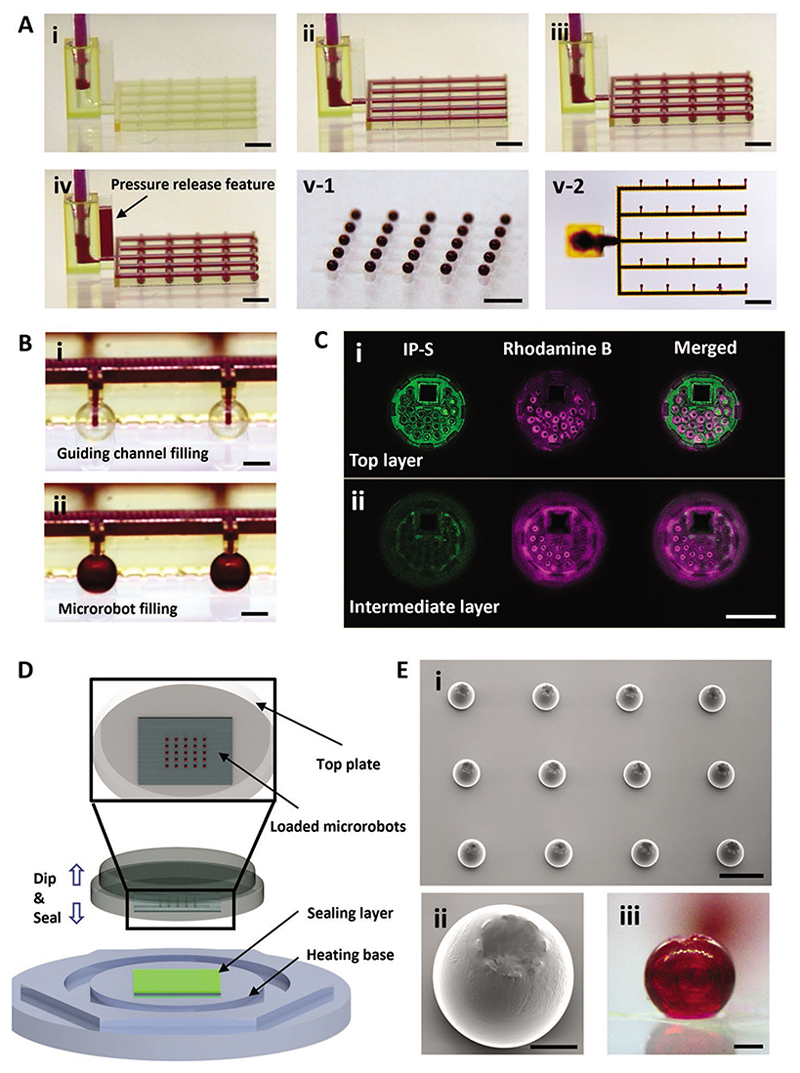
Demonstration and characterization of the MLDS system. A) The overall microfluidic loading procedure with rhodamine B solution as model cargo. i) The microfluidic channels were connected with a flexible tube. ii) The cargo was flowed through the microfluidic channels. iii) The microrobot array was fully loaded. iv) Built up pressure was released via the pressure release feature. v) The intact loaded microrobot array was separated from the microfluidic channels. Scale bar: 1 mm. B) A close view of the loading stages when cargo flows from the channel into the chambers of the microrobots. Scale bar: 200 μm. C) Confocal images of loaded microrobots: the green channel represents printed microrobot (IP-S) and the purple channel represents loaded rhodamine B solution. i) The top layer. ii) The layer at 15 μm underneath the lid. Scale bar: 100 μm. D) Schematic of the dip sealing process which consists of two parallel plates with precise motion capacity. The bottom plate has a heating function to control the melting of the sealing layer. The top movable sample holder controls the dip sealing process with minimal contact. E) i) The sealed microrobot array on the glass substrate after dip sealing using PCL. Scale bar: 500 μm. ii,iii) SEM (ii) and optical image (iii) of a single loaded and sealed microrobot on the glass substrate. Scale bars: 100 μm.

The microfluidic loading process consisted of five sequential steps (Figure 2A and Movie S2, Supporting Information): i) the microfluidic channels were first connected to a syringe pump via flexible tubing. Rhodamine B solution was used as a model cargo; ii) the cargo was perfused through the microfluidic channels and iii) into the microrobots via a guiding channel extending into the microrobot chamber; iv) excess pressure build-up during loading was relieved via the pressure escape feature. This contributed to a clean microrobot surface (Figure S6, Supporting Information), which enabled strong bonding between the microrobot sealing layer and promoted stability of the sealing layer; and v) then, the microfluidic channels were successfully separated from the microrobot array via the breakaway interface features resulting in: v-i) an intact microrobot array and v-ii) separated microfluidic channels. In particular, the guiding channel extending into the microrobot chamber facilitated loading by reducing surface tension (Figure 2B and Movie S3, Supporting Information), which was confirmed via confocal microscopy images (Figure 2C and Movie S4, Supporting Information). We also quantified cargo in small numbers of randomly selected microrobots, in order to evaluate the precision of the loading process across the arrays. Two groups each of a single microrobot and four microrobots (*n* = 3) were incubated in 400 μL phosphate-buffered saline (PBS) solution; cargo extraction was characterized via UV–Vis analysis of dye concentration in the solution. As shown in Figure S7, Supporting Information, we observed nearly identical loading across samples in each group, which indicates that the loading process is precise across the array.

The microrobots were then sealed to protect cargo and enable controlled release. We propose a “dip sealing” method where inverted microrobots briefly contact a melted polymer layer that adsorbs to the microrobot lid to form a sealant (Figure 2D). To maintain fine control over these parameters, we exploited the automated movement platform of a rheometer, which includes a temperature controllable base and contact detection. This allowed for the sealing process to be achieved with high precision. Polycaprolactone (PCL) was selected as the sealing material because it has a melting temperature above human body temperature (a mixture of PCL diol *M*_n_ 2000 g mol^−1^ and PCL *M*_n_ 525–575 g mol^−1^ in a weight ratio of 2:1, *T*_m_ ≈ 45 °C). In the first step of the dip-sealing process, the microrobot array was fixed onto the rheometer top plate. Next, a thin layer of molten PCL was spin-coated onto a glass substrate before being secured onto the heated bottom plate of the rheometer. Then, the rheometer transiently brought the top plate (microrobot array) into contact with the bottom plate (PCL layer) to precisely and robustly seal the microrobots with the PCL, which solidifies at room temperature after dipping (Movie S5, Supporting Information). Several parameters critically influenced the sealing performance, including the size of the square inlet (Figure S8, Supporting Information) and the microprotrusions around the lid; the latter increased contact area between the microrobot and the resin and controlled the thickness of the sealing layer (Figure S9A, Supporting Information). As a control, micro-robots that did not possess the protrusions were unsuccessfully sealed (Figure S9B, Supporting Information). Microrobots were subsequently confirmed to be successfully sealed and to maintain full structural integrity (Figure 2E-i,ii). Importantly, optical imaging showed that the loaded rhodamine B solution remained fully within the microrobot chambers (Figure 2E-iii and Figure S10, Supporting Information).

### 2.3 Externally Mediated Release of Cargo from MLDS-Derived Microrobots

**Figure 3:**
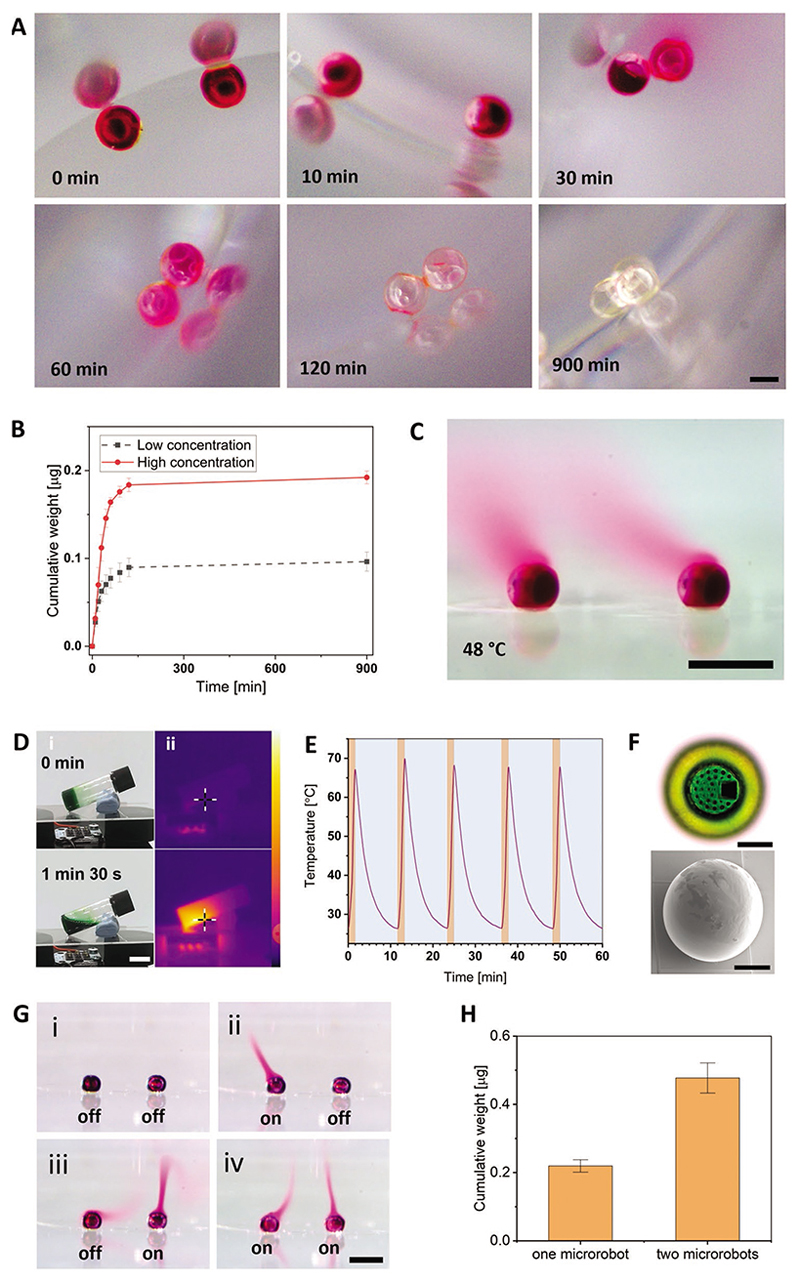
Externally mediated release of cargo from MLDS-derived microrobots. A) Optical images of the microrobot cargo release process in response to thermal energy (65 °C) over time. Scale bar: 200 μm. B) Cumulative cargo release from microrobots with two concentrations of rhodamine B (1% and 2%) following thermal triggering at 60 °C. Data shown as mean ± standard deviation (S.D.), *n* = 3. C) Real-time release of rhodamine B from microrobots on the glass substrate, triggered by increasing the environmental temperature to above the *T_m_* of the PCL sealing layer. Scale bar: 500 μm. D) Photothermal analysis of the NIR-PCL resin. i) Optical images of the phase change process (from solid to liquid) when exposed to NIR light. ii) Corresponding thermal images of the same process show the temperature distribution under NIR light. The temperature range bar is from 23 to 84 °C. Scale bar: 1 cm. E) Temperature fluctuations of the NIR-PCL resin over time under cyclic NIR irradiation. F) Top view of a single sealed microrobot with the green NIR-PCL sealing layer. SEM image of the side view of a single sealed microrobot verifies strong bonding between the NIR-PCL and the microrobot. Scale bar: 100 μm. G) The selective release of the loaded microrobots sealed by NIR-PCL. The release can be locally triggered on a single microrobot by controlling the NIR-light exposure. i,iv) Laser is switched with exposure on both microrobots. ii,iii) Laser is switched with exposure on single microrobot. Scale bar: 500 μm. H) Comparison of the release quantity from one and two microrobots. The cumulative weight released from two microrobots is twice as much as that from one microrobot. Data shown as mean ± S.D., *n* = 5.

On-demand release is a promising strategy in controlled cargo delivery, as it simultaneously maximizes the dosage at the target site and avoids systemic off-target effects. An ideal microrobotic device allowing for on-demand release in deep tissues requires external stimuli to trigger cargo release.^[24]^ Thermal stimulation has been a popular strategy to trigger cargo release in a wide range of biomedical applications.^[25]^ The melting temperature of the biocompatible PCL sealing layer chosen in this study not only enables facile dip-sealing but also presents an opportunity to exploit thermal-trigger mechanisms for on-demand cargo release. We hypothesized that the sealing layer would become cargo-permeable when heated above its melting point (Movie S6, Supporting Information). Thus, the cumulative cargo release profile was studied under a controlled thermal environment (Figure 3A). Rhodamine-loaded microrobots were initially dark red, gradually became light red during the release process, and were fully clear when cargo was completely released by 15 h. The relationship between the rhodamine B concentration and absorbance (Figures S9 and S10, Supporting Information) was used to calculate the amount of cargo released.

To demonstrate the robustness of the presented MLDS, two aqueous rhodamine B solutions (“low” 1% and “high” 2% concentration] w/v]) were loaded into the microrobots and their release was studied (Figure 3B). When thermally triggered at 60 °C, the cargo release process featured an initial burst followed by a more sustained release over time. The total cumulative rhodamine B release was correlated to the initial loading concentration (0.192 ± 0.007 μg release for 2 wt% loading versus 0.096 ± 0.011 μg for 1 wt% loading, *n* = 3 for each).

These results demonstrated that the cargoes loaded into the MLDS-derived microrobots could be adjusted in concentration and could be released in a quantitatively correlated manner. To observe the cargo release process from the microrobots in real time, a control study was conducted by increasing the temperature from room temperature to the melting temperature of PCL (Figure 3C and Movie S7, Supporting Information). In addition, SEM images indicated that cargo was released through the large loading inlet (Figure S12, Supporting Information) as the sealing layer adopted the shape of the pore array. The release profile of rhodamine B from the loaded microrobots (Figure S13A, Supporting Information) showed that the release rate was rapid in the first 30 min before reaching a plateau of 80% of the cumulative release ratio in 60 min (Figure S13B, Supporting Information).

Having established the thermal responsiveness of the PCL sealing layer for triggered cargo release, we next explored the possibility of near-infrared (NIR)-light-triggered release. NIR light can penetrate tissue with high spatiotemporal resolution to externally activate release.^[26]^ We modified the PCL by adding a NIR dye (IR-813, with a maximum absorption wavelength of 813 nm) to generate a material (NIR-PCL) that is responsive to NIR irradiation. NIR-PCL (1% w/w) became a fluid liquid resin after 30 s of NIR irradiation, with a maximum temperature (70 °C) achieved within 1 min, confirming the material’s rapid photothermal response (Figure 3D and Movie S8, Supporting Information). Moreover, alternating 1 min of irradiation (0.5 mW cm^−2^) and ≈10 min of cooling produced cyclic thermal fluctuations between 27 and 67 °C in a highly reproducible manner (Figure 3E).

In real-world applications, light would be required to penetrate deep tissues to trigger cargo release, so the responsiveness of the microrobot could be improved by higher concentrations of the NIR dye. Therefore, we employed tetrahydrofuran (THF) as a co-solvent to increase dye concentration. We then compared the photothermal properties of sealing PCL resin with 1 wt% IR-813 and 5 wt% IR-813. At higher dye concentration, the resin was much darker in color and exhibited a larger temperature increase (Figure S14, Supporting Information).

This composite resin was subsequently adopted as the sealing material to yield an improved NIR-responsive cargo release system. The co-solvent evaporated after coating on the glass substrate. Microrobots loaded with rhodamine B solution (3 wt%) were fully sealed with this NIR-PCL formulation (Figure 3F and Figures S15–S17, Supporting Information). When microrobots sealed with either pure PCL or NIR-PCL were simultaneously NIR-irradiated, only NIR-PCL-sealed microrobots were triggered to release rhodamine B whereas PCL-only-sealed microrobots remained unperturbed (Figure S18 and Movie S9, Supporting Information). The results confirmed NIR-PCL as an ideal sealing layer to conduct photothermal-triggered cargo release. In addition, we showed the spatial accuracy of NIR irradiation in triggering adjacent NIR-PCL-sealed microrobots. By alternating the irradiation between the two microrobots, alternating cargo release rates were observed (Figure 3G and Movie S10, Supporting Information). To determine whether the release of cargo could be achieved in a quantitative manner, the release amount from single and pairs of microrobots sealed with NIR-PCL was characterized (Figure S19 and Movie S11, Supporting Information). We determined that the release from 2 microrobots (0.48 ± 0.04 μg) was approximately 2 times larger than that from 1 microrobot (0.22 ± 0.02 μg), which validated the reliability of the loading and sealing process (Figure 3H). Upon irradiation, the microrobot sealed by NIR-PCL released ≈70% of its cargo within 3 min following a burst release profile. Moreover, the cargo demonstrated no leakage from the microrobots at 4 °C in PBS for at least 3 days, showing the stability with the absence of on-demand triggering (Figure S20, Supporting Information). For in vivo applications, the microrobot needs to prevent cargo release in a variety of physiological conditions before releasing the loaded cargo at the targeted site. To examine this, we first investigated cargo release from microrobots at various temperatures. The rhodamine-loaded and PCL-sealed microrobots were incubated in 200 μL PBS solution at 45 or 55 °C for 1 h. Cargo release is accelerated by around twofold at 55 °C versus 45 °C as expected. (Figure S21, Supporting Information). We also evaluated cargo encapsulation in three solutions: 37 °C simulated gastric fluid, 37 °C simulated intestinal fluid, and 37 °C PBS for 1 h. The results showed that cargo release is much lower in gastric fluid compared to elevated temperatures (around 6% of release at 55 °C, Figure S21, Supporting Information).

The properties of the sealing layer also affect cargo release. For example, responsiveness of the NIR-PCL polymer depends on both concentration of the NIR dye and the wavelength of irradiation. Two transparency windows in the NIR region have been widely used in biomedical applications: compared with the first NIR window (650–950 nm, NIR-I), the second NIR window (1000–1350 nm, NIR-II) offers deeper light penetration, higher maximum permitted exposure,^[27]^ and lower energy density. Increasing dye concentration in the NIR-PCL polymer can make up for the loss of energy density. Thus, performance of light-triggered cargo release by the NIR-PCL sealing layer can be tuned through optimization of dye concentration and NIR wavelength.

### 2.4 Applications in Drug Delivery, Environmental Sensors, and Micromotors

**Figure 4:**
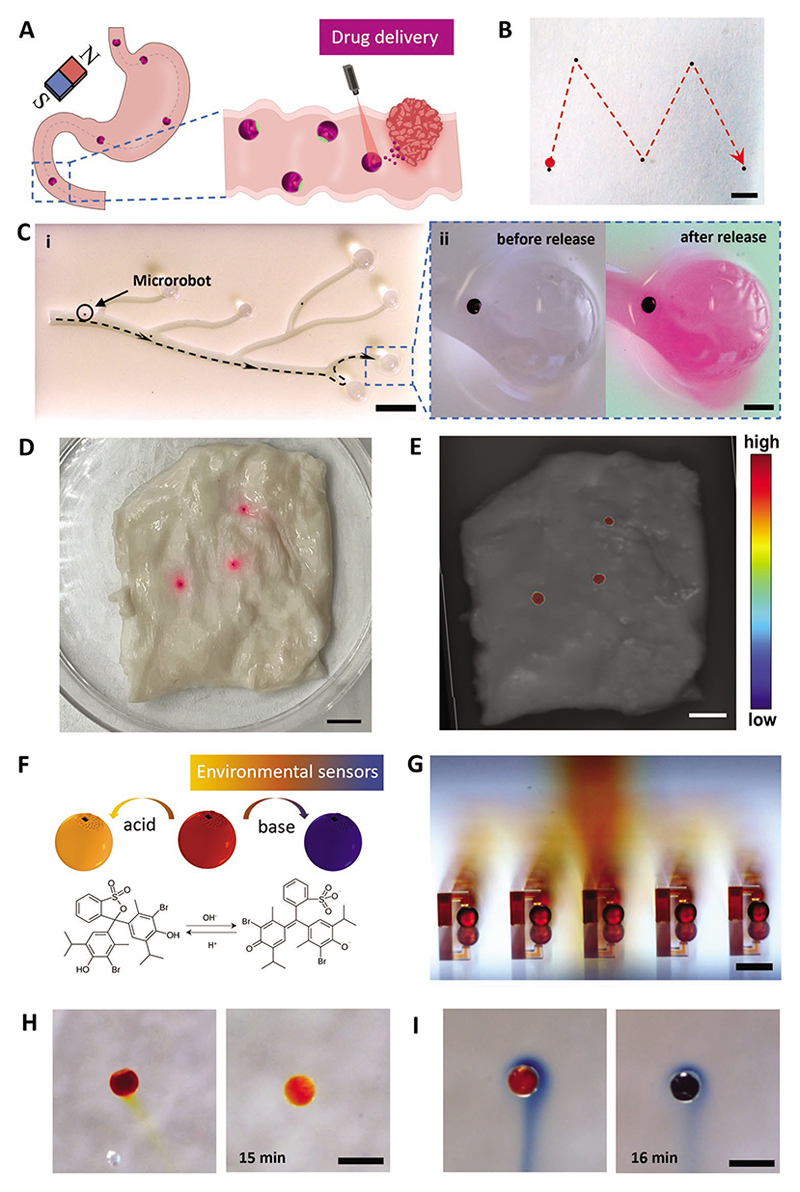
Demonstrated applications of the fillable microrobotic systems. A) Schematic of the potential targeted gastrointestinal drug delivery. B) Motion control of the microrobot in an open environment. Scale bar: 2 mm. C) i) Demonstration of targeted motion in a printed vascular-like environment. Scale bar: 5 mm. ii) Comparison before and after NIR triggered release around the target area. Scale bar: 500 μm. Evaluation of cargo release on ex vivo pig intestine tissue. Three fully loaded and sealed microrobots were distributed on the intestine surface. D) Optical image and E) fluorescence molecular tomography (FMT) of thermally triggered cargo release from these three microrobots. The color scale bar represents fluorescence signal intensity. Scale bar: 5 mm. F) Working principle of the pH sensor bromothymol blue with color changes under different pH conditions. G) The pH indicator solution is loaded into the microrobot array by the microfluidic loading technique. Scale bar: 500 μm. H) Sensing result in acidic conditions (pH = 2) demonstrating a color change from dark yellow to light yellow when the loaded indicator was released. I) Sensing result in basic conditions (pH = 13) demonstrating a color change from dark yellow to blue when the loaded indicator was released. Scale bar: 500 μm.

Beyond on-demand triggered cargo release mediated by the sealing layer, magnetically imbued microrobots could be further actuated by external magnetic fields to realize a targeted drug delivery (Figure 4A). This has become a popular strategy as magnetic fields are capable of high resolution and noninvasive manipulation of microscale objects within the human body.^[28]^ This is especially important in clinical applications requiring minimal off-target drug accumulation or reduced side effects (Scheme 1).

To demonstrate this with the MLDS system, we sequentially coated nickel (to facilitate magnetic responsive behavior) and titanium (to improve biocompatibility)^[13b]^ onto the microrobot surface to facilitate precise actuation under external magnetic fields. Importantly, the spherical shape of the microrobot facilitates a rolling motion that is advantageous in pipe-like environments such as blood vessels and the gastrointestinal tract.^[29]^ Under an externally applied magnetic field, the microrobots could move in a fluidic solution along a predetermined path (Figure 4B) and within a complex architecture involving walled channels and tunnels in the shape of “ICL” (Figure S22A and Movie S12, Supporting Information). To further showcase the capabilities of MLDS microrobots in targeted on-demand cargo delivery, we constructed a complex branching vascular-like structure with tumor-mimicking alginate phantoms as a model target site (Figure 4C). Within this structure, the microrobots could roll along the wall in response to the externally applied magnetic field (Figure S22B and Movie S12, Supporting Information). This level of fine motion control is especially beneficial when navigating complex vascular environments and permits course correction when moving between different vessel branches (Movie S13, Supporting Information). Upon reaching the target sites (alginate spheres), NIR irradiation triggered the release of the rhodamine B cargo for on-demand delivery, which was confirmed by a color transition of the alginate sphere. This experiment further demonstrated the high performance of the microrobot system for targeted drug delivery in terms of magnetic motion control, efficient cargo loading, and on-demand release. Further, the size of the microrobot produced by MLDS can be adjusted to meet the requirements of different application areas; for example, blood vessels would require much smaller microrobots compared to the gastrointestinal tract. A range of sizes (165, 310, and 495 μm) have been fabricated to validate the size tunability of the MLDS platform (Figure S23, Supporting Information). Collectively, these properties highlight the potential of the MLDS system for various clinical drug delivery applications.

We also evaluated the biocompatibility of the microrobot using cultured human mesenchymal stem cells (hMSCs).^[30]^ hMSCs are multipotent stromal stem cells that can differentiate into cells belonging to the endoderm, including gut epithelial cells,^[31]^ and have shown tremendous potential in therapy, such as vascular regeneration^[32]^ and bone regeneration.^[33]^ Microrobots made with IP-S and IP-S coated with Ni/Ti were incubated on cell monolayers for three days; subsequent LIVE/DEAD staining showed no difference between treated and untreated cells (Figure S24, Supporting Information).

To further evaluate the potential of the microrobot for drug delivery, we evaluated the biodistribution of cargo release in ex vivo pig intestine tissue. Three fully loaded and sealed microrobots were distributed on the surface of the tissue. Cargo release was locally triggered by heat, and the subsequent distribution was monitored visually (Figure 4D,E). Cargo release was concentrated near the microrobots suggesting suitability for targeted delivery applications.

It is clear that a single microrobot cannot easily provide enough of a drug for therapeutic functions. For example, selfassembly is widely used for nanoparticle systems to improve disease treatment through aggregation at the targeted site.^[34]^ Thus, it is essential to investigate the control of multiple microrobots within the body. Inspired by the fascinating collective intelligence of natural living organisms, various artificially “swarming” robots have been reported.^[35]^ Compared with independent microrobots with small sizes and volumes, the swarms are expected to maintain higher loading capability and demonstrate controllable patterns during locomotion with proper global input. As magnetic swarm actuation and control strategies are now well-established,^[36]^ we suggest that microrobots fabricated by the MLDS method could be controlled in such a swarm. Furthermore, since the MLDS strategy allows for discrete adjustment of loading solutions, it may be used to assemble a heterogeneous swarm composed of microrobots with specialized functionalities.

Beyond drug delivery, the MLDS system may also provide opportunities in various applications including environmental sensing or chemically powered micromotors. This versatile potential can be attributed to the ability of the MLDS system to load various types of fluidic cargoes into microrobots. Bromothymol blue (BB) is a widely used pH indicator with obvious color change when transitioning from yellow at pH = 6.0 to blue at pH = 7.0.^[37]^ To demonstrate proof-of-concept pH sensing, we used the MLDS platform to load BB dimethyl sulfoxide (Figure 4F) into microrobots and seal the microrobots with PCL (Figure 4G and Movie S14, Supporting Information). When the microrobot was stimulated by heat, the sealing layer melted, which partially released the cargo into the surrounding solution and produced a color transition starting from the lid of the microrobot (Figure 4H). After 15 min, the surrounding acidic solution infused into the chamber of the microrobot and changed the color of the microrobotic structure. In addition, a reciprocal test was performed by immersing the loaded microrobot into an alkaline solution; upon temperature-mediated cargo release, the BB was deprotonated and displayed the expected color change to blue (Figure 4I). The sensing accuracy of BB-loaded microrobots was further evaluated in a series of solutions with different pH levels: pH 6, 8, and 10 solutions and PBS. Fully loaded microrobots were placed into 300 μL of each solution, which exhibited the expected changes in color and absorbance wavelength (Figure S25, Supporting Information).

Our MLDS system also showed potential for the preparation of micromotors with promising applications in therapeutic treatment. The principle behind chemically powered micromotors is based on the implementation of self-propelling materials. These materials are capable of catalyzing the conversion of chemical fuels to gas-forming species that ultimately produce motion.^[35a]^ One classic example is the use of hydrogen peroxide as fuel which, in presence of peroxidative catalysts, generates oxygen bubbles that act as propellants.^[38]^ To exploit this system, we loaded a catalyst cargo solution composed of aqueous iron (III) nitrate into MLDS microrobots (Figure S26A, Supporting Information) and immersed them into a hydrogen peroxide solution. Upon thermally triggered cargo release, the iron catalyst in the microrobot was oxidized by hydrogen peroxide, rapidly increasing the pressure of oxygen gas and propelling the microrobot (Figure S26B and Movie S15, Supporting Information). Beyond demonstrating cargo protection and triggered release, this experiment confirmed that chemical propulsion can complement magnetic actuation in future studies.

## 3 Conclusion

Using the MLDS platform we can manufacture fillable microrobot systems to provide new possibilities for biomedical microrobotic systems. The fillable chamber design isolates the loaded cargo from its surroundings, maintains cargo stability, and facilitates high loading capacity. Importantly, the loading process is direct, precise and simple, which improves loading efficiency and minimizes waste without requiring additional chemical modification of the cargoes. The dip sealing method efficiently protects the cargo encapsulated within the microrobot and is adaptable to various materials that facilitate different on-demand release mechanisms, including thermal and photothermal pathways. Using this platform, we loaded different cargoes for various applications including drug delivery, pH-responsive environmental sensing, and chemically powered micromotors.

Compared to existing methods, our system offers four key advantages: i) high cargo loading requiring low stock volumes with minimal waste, which is ideally suited for costly cargo; ii) a cargo-agnostic loading technique which offers full protection within the hollow chamber against external environmental factors; iii) elimination of chemical coupling and processing conditions during loading, which might degrade sensitive cargoes; and iv) inclusion of smart material layers for stimuli-responsive functionality through the dip sealing process. MLDS is a platform technology that expands opportunities in microrobotics for targeted on-demand cargo release and biosensing with an all-in-one fabrication method.

## 4 Experimental Section

### Materials

All chemical reagents were used without further purification. IP-S (Nanoscribe) was used as the printing resin. Acetone (VWR 20066.330) and pure water (Millipore Milli-Q Integral 3) were used to clean the substrate. PGMEA (Sigma-Aldrich 484431) and isopropanol (IPA, Fisher Chemical P/7500/17) were used to remove the non-polymerized resin. ITO coated glass (Nanoscribe) was used as the printing substrate. 3-(trimethoxysilyl)propyl methacrylate (Sigma-Aldrich 440159) and ethanol (VWR 20821.330) were used for surface treatment of ITO glass. Rhodamine B (Sigma-Aldrich R6626) was used as loading cargo for the drug release demonstration. PBS (Gibco DPBS,1X) was used for release studies. Polycaprolactone (PCL) diol (*M*_W_ 525–575, FP45122 Biosynth Carbosynth) and polycaprolactone diol (*M*_n_ ≈2000, Sigma-Aldrich 189421) were used to optimize thermoresponsive PCL resin formulations. IR-813 p-toluenesulfonate (TCI C2886) was used as a NIR-responsive dye for NIR-triggered drug release (NIR-PCL resin) experiments. Tetrahydrofuran (THF, VWR 28551.321) was used as the NIR-PCL solvent. Bromothymol blue (Sigma-Aldrich 114413) and dimethyl sulfoxide (DMSO, VWR 23500.260) were used as pH indicator and solvent, respectively. Hydrogen peroxide (Sigma-Aldrich 216763) and iron(III) nitrate (Sigma-Aldrich F8508) were used as a chemical fuel and catalyst for chemically powered micromotor experiments. Methylene blue (Sigma-Aldrich M9140) was used as a color indicator. hMSCs (Lonza, PT-2501) were used for cell viability study. alamarBlue Cell Viability Reagent (ThermoFisher, DAL1025), LIVE/DEAD Viability/Cytotoxicity Kit (ThermoFisher, L3224), MesenPRO RS Medium (ThermoFisher, 12746012), and Trypsin-EDTA (0.05%) (ThermoFisher, 25300054) were used for cell viability study. Buffer solutions (pH 6.0, pH 8.0, and pH 10.0, Sigma-Aldrich) were used for characterization of pH sensing accuracy.

### Substrate Preparation

ITO-coated glass was used as the substrate onto which the integrated microfluidic structure was printed. The resistance of the glass surface was assessed with a multimeter (Amprobe PM51A) to confirm the ITO-coated side. The ITO coating was necessary for increasing the refractive index contrast, to find the printing interface between the resin and the substrate. The substrate was cleaned by rinsing with acetone, IPA and water sequentially to remove organic substances and dust, then dried with nitrogen followed by for 5 min bake at 120 °C. The printed structure required strong adhesion bonding with the substrate to prevent damage during the manual operation of connecting the microfluidic platform with an external syringe via flexible tubes. To improve the adhesion between the substrate and printed structure, a surface treatment with silanization was required. The ITO-coated surface of the cleaned glass was activated by O_2_ plasma (GaLa Instrumente Prep 5, 0.3 bar, 150 W) for 5 min to make it hydrophilic. The plasma-treated glass was then immersed into a solution of ethanol and 3-(trimethoxysilyl)propyl methacrylate (at a ratio of 1/5 v/v) for 2 h. The treated glass was rinsed with acetone and dried with nitrogen before use.

### Printing, Cleaning, and Post-Processing

The smallest features of the microfluidic platform, with point-to-point distances of less than 10 μm, required a high-resolution printing technique to fabricate. To manufacture the system, two-photon polymerization (2PP) was chosen which is a direct 3D writing process employing a near-infrared femtosecond laser that provides enough light intensity to trigger the polymerization of a photosensitive resin. Therefore, a commercial 2PP printer (Photonic Professional GT2, Nanoscribe) was used to print the microfluidic structure using the commercial resin IP-S. The model was designed in a commercial computer-aided design software (Autodesk Inventor 2022) and its model file (STL) was exported. The model file was processed in a job preparation program (Describe 2.7, Nanoscribe), whereby a shell and scaffold printing technique was optimized to achieve a reasonable printing time while maintaining high mechanical strength. The preparation program divided the print into two parts including shells and interior scaffolds. Outer shells were required for structural features, whilst interior scaffolds maintained mechanical stability without increasing the printing time significantly. The optimized parameters included: slice distance 1 μm, hatch distance 0.5 μm, shell contour count 12, base slice count 8, shellLaserPower 90, shellScanSpeed 100 000, scaffoldLaserPower 90, scaffoldScanSpeed 100 000, block size *x* 500 μm, *y* 500 μm, *z* 290 μm, block overlap 8, layer overlap 3.

For printing, the previously treated ITO glass was placed into its dedicated holder, and the IP-S resin was applied to an amount sufficient for printing. Vacuum degassing was employed to eliminate bubbles from the resin. Under a 25× NA0.8 objective, the printing process typically lasted 12 h. After printing, the cured IP-S structure remained immersed within the uncured resin, which also filled the microfluidic channels within the structure. To remove uncured resin from the hollow microfluidic channels, micropores were implemented along the channels to promote resin removal by diffusion. To do this, the entire substrate was submerged into a beaker with PGMEA, positioned with the printed structure facing down, and magnetically stirred at 200 rpm and 45 °C for 3 h.

Subsequently, the entire substrate was submerged in IPA for 1 h to remove excess PGMEA. Once fully cleaned, the substrate was dried using nitrogen and post-cured under UV light (Form Cure *λ* = 405 nm) for 2 h.

### Imaging Characterization

To verify that the detailed patterns of the MLDS platform were well fabricated, the printed structures were sputter-coated with 200 Å Au and imaged by scanning electron microscopy (JEOL 6010LA).

### Microfluidic Loading and Dip Sealing

The microfluidic loading was achieved by a controllable process using a syringe pump (Chemyx Fusion 100). The printed structure was designed with an external connection port (inner diameters tapered from 800 to 570 μm over 1700 μm) that was connected to a syringe (BD Micro-Fine 0.5 mL 037–7614) via a flexible PTFE tube (Bohlender GmbH S1810-04, outer diameter of 0.6 mm).

Solutions of rhodamine B in PBS at various concentrations (1 wt%, 2 wt%, and 3 wt%) were used as model loading cargoes. To load the cargoes, a syringe pump flow rate of 0.0025 mL min^−1^ was used to infuse the cargo through the syringe, tube, microfluidic channels, and simultaneously fill the chambers of each microrobot. The loading process was recorded by a camera (Canon EOS 700D, Movie S2 and S3, Supporting Information). Upon loading completion, the PTFE tube was separated from the connection port whilst the structure remained adhered onto the substrate.

Before sealing, the loaded microrobots were kept in a 4 °C fridge to avoid cargo evaporation. The dip sealing method was developed to introduce a thin layer covering the lid of microrobots, protecting the encapsulated cargo from leaking or premature exposure to the surrounding environment. The sealing layer was prepared by spincoating the sealing resin onto a glass substrate, which was placed on the base of a rheometer (Anton Paar Modular Compact Rheometer MCR 302) to promote a mechanically controllable process. The base was heated to 60 °C to maintain resin fluidity. The substrate containing a loaded microrobot array was fixed onto the top parallel plate, which was mechanically lowered onto the sealing resin to complete one cycle of the dip sealing procedure (Movie S5, Supporting Information). This contact immersed the lid of the microrobots into the sealing resin, and the process was monitored by the rheometer with a low contact force of less than 2 N to avoid breaking the microrobots. Once contact with the base was detected, the top plate moved upward and a thin layer of melted PCL resin would stick to the microrobot surface to seal the openings on the lid.

A complete dip sealing process consisted of four cycles to achieve a reliable sealing layer. Each successful cycle was verified by visual confirmation of an imprint left on the sealing substrate.

### Thermoresponsive Sealing Resin

PCL was used as the sealing material due its suitable melting temperature and excellent biocompatibility. Considering the requirement for biomedical applications, the melting temperature of the PCL sealing layer should be optimized to be slightly above 37 °C. Two types of PCL (PCL *M*_n_ = 525 g mol^−1^ and PCL *M*_n_ = 2000 g mol^−1^) was mixed in a weight ratio of 2:1 to achieve a composite with a melting point of around 45 °C.

To form a thin layer of thermo-responsive PCL, a glass substrate was first treated with O_2_ plasma for 5 min. Then, the optimized PCL resin formulation was melted, and whilst maintaining a temperature above the melting temperature of the resin, the glass substrate was spin-coated (Laurell WS-650SZ-6NPP) at 2000 rpm, 500 ramp and for 30 s.

### NIR-Light-Responsive Sealing Resin

To provide the resin with light-responsive properties, IR-813 was blended into the thermo-responsive PCL resin described above. For a low dye concentration (1 wt%), melted PCL resin was directly mixed with the dye. For a high concentration dye (5 wt%), melted PCL resin was first mixed into THF, before adding IR-813 to a final weight ratio of PCL/THF/IR-813:1/1/0.05.

To form a thin layer of NIR dye-blended PCL (NIR-PCL), a glass substrate was first treated with O_2_ plasma for 5 min and then spin-coated with optimized resin at 1000 rpm, 500 ramp, and for 30 s (Figure S15, Supporting Information).

### Cumulative Release Study in Thermal Release

5 loaded and sealed microrobots were removed from the printing substrate and used as one group. The microrobots were placed in one well (96 well, Thermo Scientific 165305) and with 200 μL of PBS solution. The well plate was placed inside a thermomixer (Eppendorf ThermoMixer C) offset at 300 rpm and 60 °C. Eight time points were selected during the thermal release process including 10 min, 20 min, 30 min, 45 min, 60 min, 90 min, 120 min, and 15 h. Images were taken at each time point (Canon EOS 700D).

### Controlled Release Study in Thermal Release

Loaded and sealed microrobots that were adhered on the substrate were submerged into PBS. The substrate was placed on a hotplate with temperature changes monitored using a thermometer (OMEGA HH306A). The release process was recorded by a camera (BASLER puA2500-14uc, Lens C23-3520-5M-P f35mm).

### Photothermal Efficiency in NIR Release

The photothermal properties of NIR-PCL were studied. NIR-PCL (1 wt% dye) was illuminated using an LED array (OSLON ILR-IO09-85SL-SC211-WIR200). The distance between the sample and the light source was 12 mm with a power density 0.5 W cm^−2^. Temperature changes of the material during NIR exposure were monitored using a thermal imaging camera (FLIR ONE).

### NIR-Triggered Release

A NIR laser (808 nm IR InfraRed at 0.6 W cm^−2^) was used to trigger the release of loaded cargo from NIR-PCL sealed (5 wt% dye) microrobots.

### Selective Release

Two loaded and sealed microrobots adhered on printing substrate were selected. The substrate was submerged into the μ-Slide well (IBIDI) with PBS. Cargo release was initiated by the NIR laser onto the microrobot (melting the NIR-responsive sealing layer), whilst cargo release reduced upon removal of the NIR laser (causing sealing layer resolidification).

### Thermal-Release Measurement

At each time point, PBS solution with release cargo was gently aspirated out from the well whilst avoiding contact with the microrobots. Then, fresh PBS (200 μL) was added into the same well. The release process was continued until the next time point. At each time point, the amount of released cargo was measured based on UV–Vis spectroscopic absorbance measurements against the standard curve of known concentrations (Figure S11, Supporting Information).

### NIR-Release Characterization

Loaded microrobots were removed from the printing substrate and combined with 400 μL of PBS solution. Microrobots immersed in PBS were repeatedly exposed to cycles of NIR irradiation, with absorbance measurements used to monitor the cargo release. The PBS solution was measured to calculate the released cargo based on UV–Vis spectroscopic absorbance measurements against the standard curve of known concentrations (Figure S11, Supporting Information).

### Simulated Gastric Fluid (SGF) and Simulated Intestinal Fluid (SIF)

SGF and SIF were prepared following a published protocol for producing an in vitro digestion environment.^[39]^ First, various electrolyte stock solutions were prepared: 37.3 g L^−1^ KCl, 68 g L^−1^ KH_2_PO_4_, 84 g L^−1^ NaHCO_3_, 117 g L^−1^ NaCl, 30.5 g L^−1^ MgCl_2_(H_2_O)_6_, and 79 g L^−1^ (NH_4_) HCO_3_. SGF and SIF were then prepared by mixing the stock solutions and adjusting the pH. SGF was composed of 7.8 mmol L^−1^K^+^, 72.2 mmol L^−1^ Na^+^, 70.2 mmol L^−1^ Cl^−^, 0.9 mmol L^−1^ H_2_PO_4_^−^, 25.5 mmol L^−1^ HCO_3_^−^, 0.1 mmol L^−1^ Mg^2+^, 1.0 mmol L^−1^ NH_4_^+^ and 0.15 mmol L^−1^ Ca^2+^, and had a of 3. SIF was composed of 7.6 mmol L^−1^ K^+^, 123.4 mmol L^−1^ Na^+^, 55.5 mmol L^−1^ Cl^−^, 0.8 mmol L^−1^ H_2_PO_4_^−^, 85 mmol L^−1^ HCO_3_^−^, 0.33 mmol L^−1^ Mg^2+^ and 0.6 mmol L^−1^ Ca^2+^, and had a pH of 8.

### Targeted Motion and On-Demand Cargo Release

For magnetic microrobotics applications, 800 nm thickness nickel (Ni) was first deposited onto the microrobot surface to impart magnetic properties, followed by deposition of 50 nm thickness titanium (Ti) to improve biocompatibility by sputter coating (HEX Korvus Technology). A magnetic field generating system (MFG-100-I, Magnebotix) was employed to create tuneable rotating magnetic fields, providing control of the microrobot motion.

To demonstrate maneuverability of magnetic microrobots under external magnetic fields (Figure 4A and Figure S22, Supporting Information), channel structures were first printed using a commercial resin (Tough White, Prusa) with a masked stereolithography printer (MSLA SL1S, Prusa). A single microrobot was removed from the printing substrate and placed within the channel testing structure. The entire system was then positioned in the center of the magnetic field generating (MFG) device under a dynamic magnetic field (varying frequency 5 Hz, field magnitude 20 mT). The magnetic field was dynamically tuned to control the microrobot motion whilst navigating through the channel test structure. The target location was an alginate sphere, whereby successful navigation of the magnetic microrobot into the target location was followed by NIR irradiation to release the cargo (aqueous rhodamine B) into the alginate sphere.

### Seeding of hMSCs

hMSCs (Lonza, passage 5) cultured in a T175 flask were washed with PBS, detached with 0.05% (w/v) trypsin, and transferred from the flask with Mensen Pro 2 medium. Cells were pelleted by centrifugation, resuspended in Mensen Pro 2 medium, and seeded at 20k cells/well in a 96 well plate.

### Cell Viability

Two days after cell seeding, microrobots were UV sterilized for 30 min and then added to the wells seeded with hMSCs. Microrobots were incubated with the cells for three days under standard culture conditions. The alamarBlue Cell Viability Reagent (ThermoFisher) assay was performed after 24 and 48 h of incubation according to the manufacturer’s instructions. LIVE/DEAD staining was performed after 72 h of incubation using the LIVE/DEAD Viability/Cytotoxicity Kit (ThermoFisher).

### Ex Vivo Demonstration

The biodistribution of cargo release was evaluated in an ex vivo tissue model. Fully loaded and sealed microrobots were placed on pig intestine obtained from a local abattoir and cargo release was thermally triggered. A fluorescence tomography system (FMT 4000, PerkinElmer, Inc., USA) was used for fluorescencebased imaging following excitation at 790 nm.

### Environmental Sensing

pH levels could be used as an indicator of environmental conditions. In this work, in situ pH sensing was demonstrated using the presented platform (Figure 4D). The microfluidic loading system was versatile for loading various types of liquids. Thus, 1 g of bromothymol blue was dissolved in 2 mL of DMSO as a pH indicator solution. This solution was then loaded into the microrobot using the microfluidic loading process. Then, the loaded microrobots were removed from the substrate and placed inside solutions of pH2 and 12, respectively. Thermal heating of the solution triggered the release of the pH indicator solution from the microrobots, subsequently changing the color of the solution.

### Chemically Powered Micromotor

To demonstrate the ability of microrobots to act as micromotors, a chemical reaction was adopted to provide the propelling motion. Hydrogen peroxide was used as the chemical fuel, which was converted to oxygen bubbles via Fenton catalysis (Figure 4H). The catalyst solution was prepared using 7.5 g iron(III) nitrate added to 4 mL of pure water. 50 mg of methylene blue was added into 2 mL of the catalyst solution to darken the color and increase visual detection. A similar microfluidic loading process was applied to load the catalyst solution into microrobots. Then, loaded microrobots were placed into 10 wt% hydrogen peroxide solution. The reaction was initiated by thermal heating, triggering the release of the catalyst solution from the loaded microrobots into the hydrogen peroxide, thereby generating bubbles that propelled the microrobots.

### Statistical Analysis

All statistical analyses were performed using the IBM Statistical Package for the Social Sciences (v.26, SPSS, Chicago, IL, USA). Results were expressed as the mean ± standard deviation (*n* = 3). All statistical comparisons were made using one-way analysis of variance (ANOVA) followed by Dunnett’s test. A difference with a *p*-value of less than 0.05 was considered statistically significant. “ns” represents *p* > 0.05, *p ≤ 0.05, **p ≤ 0.01.

## Supplementary Material

Supplementary materials

## Data Availability

The data that support the findings of this study are openly available in Zenodo at https://doi.org/10.5281/zenodo.7409235.
