# Supplementary material for: Assembly of Fillable Microrobotic Systems by Microfluidic Loading with Dip Sealing
Source: Adv Mater. Author manuscript; Available in PMC 2024 Jan 5. (PMC7615483; doi:10.1002/adma.202207791)
Supplement: Supplementary materials [file EMS175908-supplement-Supplementary_materials.pdf]

ADVANCED MATERIALS

Supporting Information

for *Adv. Mater.,* DOI: 10.1002/adma.202207791

Assembly of Fillable Microrobotic Systems by Microfluidic Loading with Dip Sealing

*Rujie Sun, Xin Song, Kun Zhou, Yuyang Zuo, Richard Wang, Omar Rifaie-Graham, David J. Peeler, Ruoxiao Xie, Yixuan Leng, Hongya Geng, Giulia Brachi, Yun Ma, Yutong Liu, Lorna Barron, and Molly M. Stevens**

Assembly of fillable microrobotic systems by microfluidic loading with dip sealing

*Rujie Sun[1]†, Xin Song[1]†, Kun Zhou[1], Yuyang Zuo[2], Richard Wang[1], Omar Rifaie-Graham[1], David J. Peeler[1], Ruoxiao Xie[1], Yixuan Leng[1], Hongya Geng[4], Giulia Brachi[1], Yun Ma[1], Yutong Liu[5], Lorna Barron[1], Molly M. Stevens[1,2,3]**

[1]Department of Materials, Imperial College London, London SW7 2AZ, UK

[2]Department of Bioengineering, Imperial College London, London SW7 2AZ, UK

[3]Institute of Biomedical Engineering, Imperial College London, London SW7 2AZ, UK

[4]Institute of Biopharmaceutical and Health Engineering, Tsinghua Shenzhen International Graduate School, Tsinghua University, Shenzhen 518055, China

[5]Department of Metabolism, Digestion and Reproduction, Imperial College London, London SW7 2AZ, UK

***Corresponding author. Email: m.stevens@imperial.ac.uk**

†These authors contributed equally to this work

Design considerations

Pressure-release chamber

The pressure-release chamber consisted of a two-layer reservoir: (lower) an array of 20 µm pores connecting the fluidic loading channels; and (upper) an array of 8 µm pores exposed to atmospheric pressure. In the absence of this chamber, cargo loading into microrobots by the microfluidic platform caused cargo leakage (Figure S5).

Reduced contact area

Contact between the bottom of microfluidic channels and the glass substrate was optimised to enable mechanical perturbation-based separation whilst supporting the microfluidic loading process. This was achieved when the contact area was reduced from full contact to 6 % (Figure S4B).

Mechanical perturbation separations

The microrobots must be separated from the MLDS platform after loading and sealing. The separation is based on the sequential breakaway of two interfaces by mechanical perturbation. The first interface is between the microfluidic channels and microrobots, and the second one is between the microrobots and the support base. The detailed procedures are (Figure S3):

i. After loading, the microfluidic channels are held manually by tweezers. Mechanical forces are applied to separate the whole part from the microrobot array and the printing substrate.

ii. Following dip sealing, we use the tiny tip of a glass Pasteur pipette to gently touch the side of the microrobot. This process separates it from the supporting base and the printing substrate without damaging the printed structure.

Cargo release study

Standard curve between Rhodamine concentration and absorbance

A dose-response curve was obtained to correlate the cargo concentration with absorbance intensity. The curve was obtained by preparing a series of 5 rhodamine solutions with concentrations from 1 µg·ml^{-1} to 5 µg·ml^{-1}. The corresponding absorbance was measured by UV–visible spectrophotometry (Molecular Devices SpectraMax M5) at λ = 555 nm. A linear curve (r^2 = 0.1725) was fitted to the data, yielding the following formula:

$$y = 0.2249x + 0.0123 \tag{1}$$

where x is the rhodamine concentration (µg·ml^{-1}), and y is the absorbance value.

To validate the above equation, another 4 rhodamine solutions (0.4 µg·ml^{-1}, 0.8 µg·ml^{-1}, 3.5 µg·ml^{-1}, 6.0 µg·ml^{-1}) were evaluated, which showed agreement with the fitted curve (Figures S8-S9).

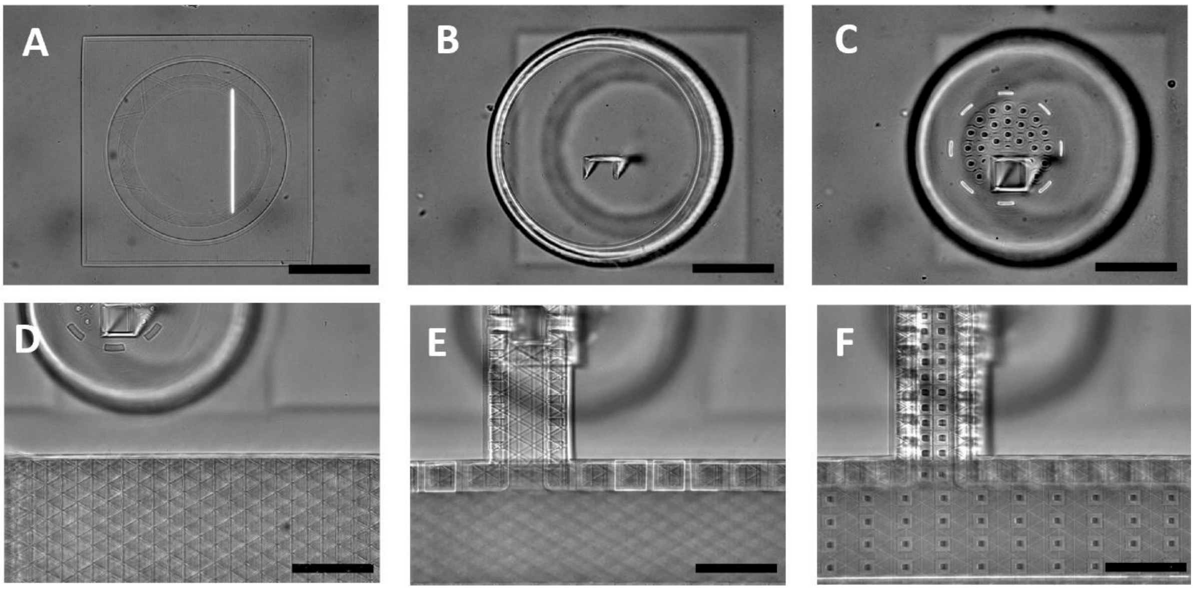

Figure S1. Fabrication of the microfluidic platforms using a 2PP-based 3D printer (Nanoscribe). (**A**) Supporting base layer integrated with the bottom of the microrobot. (**B**) The inside of the microrobot with the guiding channel in the centre. (**C**) Top features of the microrobot: an inlet for cargo loading, with micropores and protrusions along the periphery. Three stages of the connection between the microrobot and channel during printing: (**D**) the channel base, (**E**) the connection bridge between the channel and the microrobot, and (**F**) the micropores on the top of the channel. Scale bar: 100 µm.

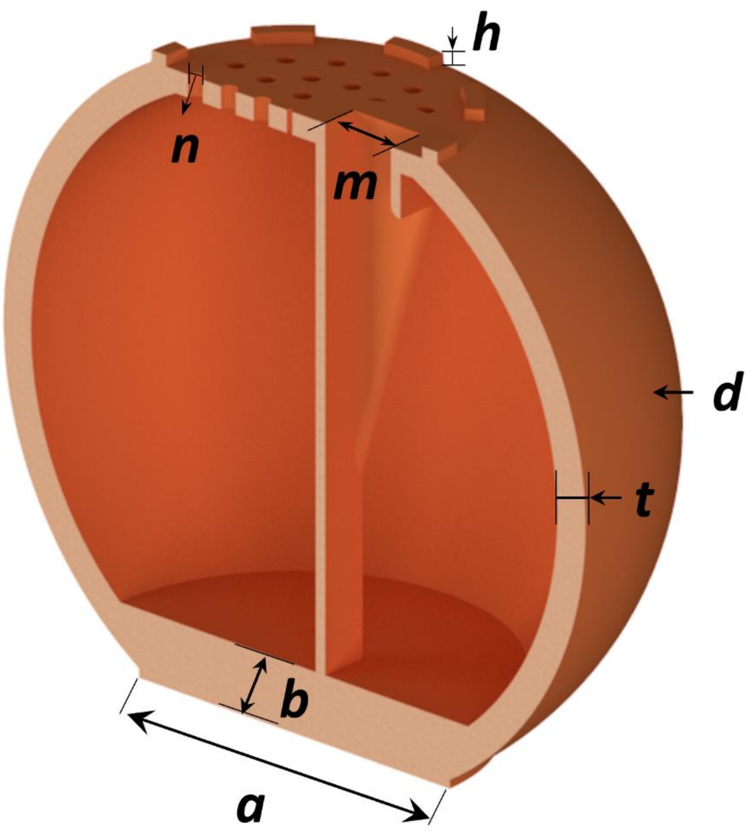

Figure S2. Microrobot geometry. The base diameter ***a*** and thickness ***b*** are 165 µm and 30 µm. The shell thickness ***t*** is 15 µm and the outer diameter ***d*** is 310 µm. The inlet edge length ***m*** is 35 µm. The diameter of the micropores ***n*** is 8 µm. The height of the protrusion ***h*** is 5 µm.

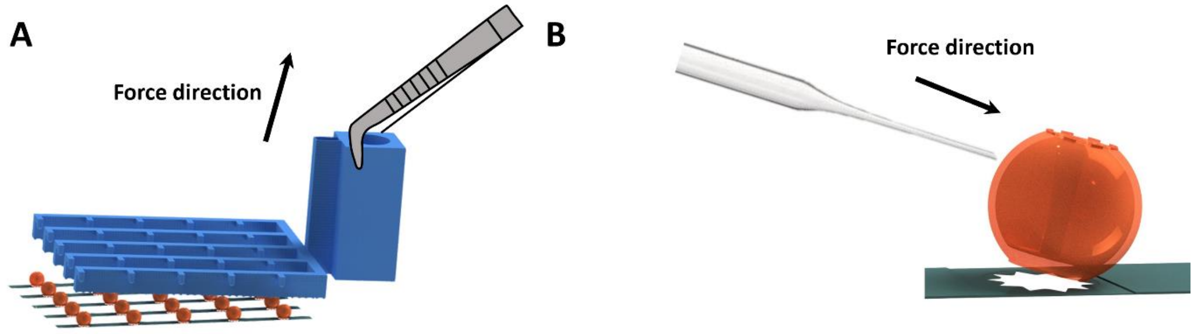

Figure S3. The detailed procedure to separate subsystems – microfluidic channels, microrobot array, and supporting base – via mechanical perturbation. (**A**) The separation between the microfluidic part and the microrobot array. Tweezers are used to apply the mechanical perturbation. (**B**) The separation between the microrobot and the supporting base. The tip of glass Pasteur pipette gently touches the side of the microrobot with a weak pushing force.

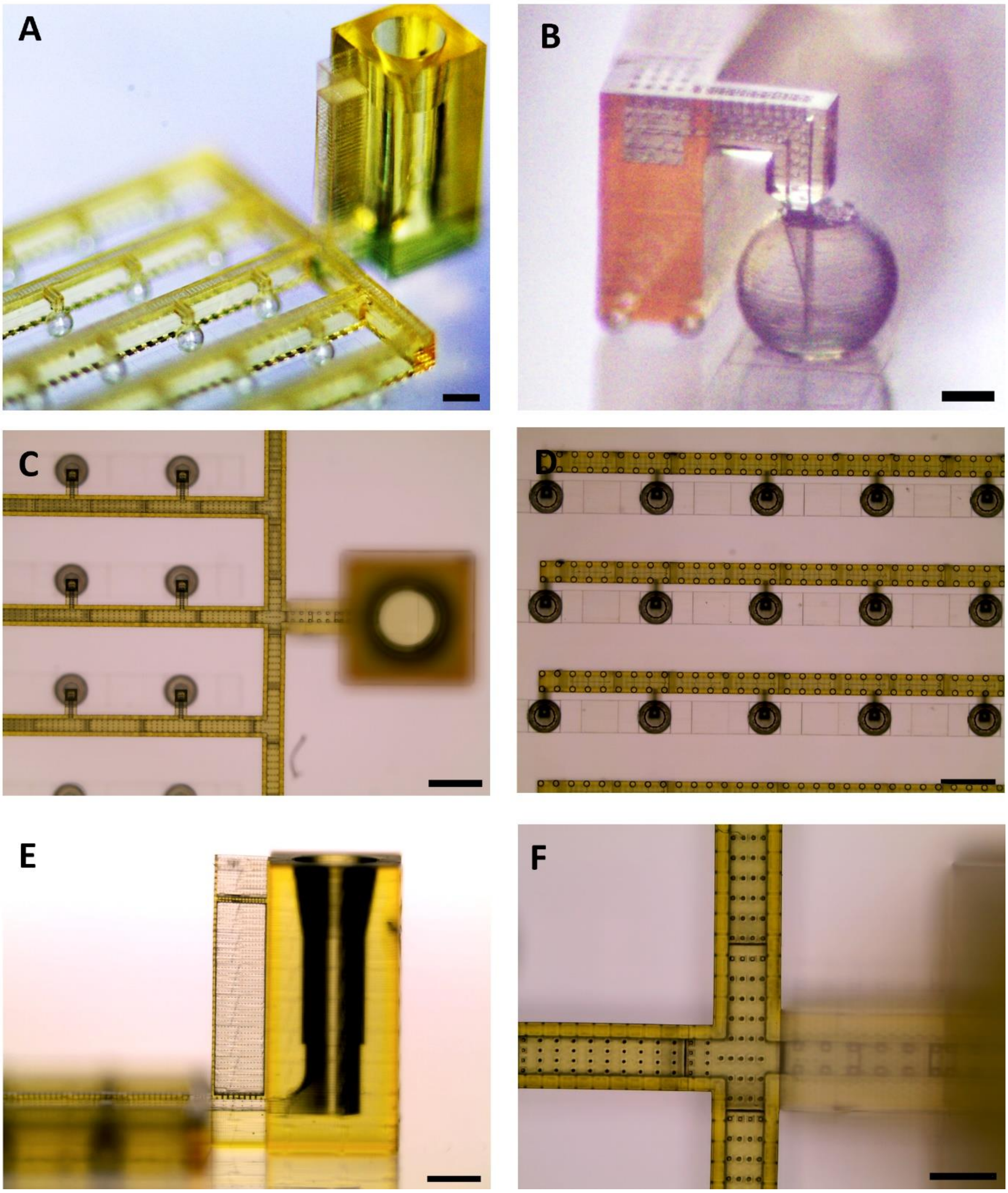

Figure S4. Optical images of the printed microfluidic platform. (**A**) An overview of the entire microfluidic loading system. Scale bar: 500 µm. (**B**) Side-view of the microrobot with the guiding channel extending into its body. Scale bar: 100 µm. (**C**) Top view of the microfluidic platform with distributed pores along the channels. Scale bar: 500 µm. (**D**) Bottom view of the microfluidic platform. A reduced contact area can be observed between the microfluidic channel and the glass substrate. Scale bar: 500 µm. (**E**) Side view of the microfluidic loading system with clear channels inside the structure and the pressure relief chamber. Scale bar: 500 µm. (**F**) Detailed view of different pore sizes along the main channel (8 µm) and pressure relief chamber (20 µm). Scale bar: 200 µm.

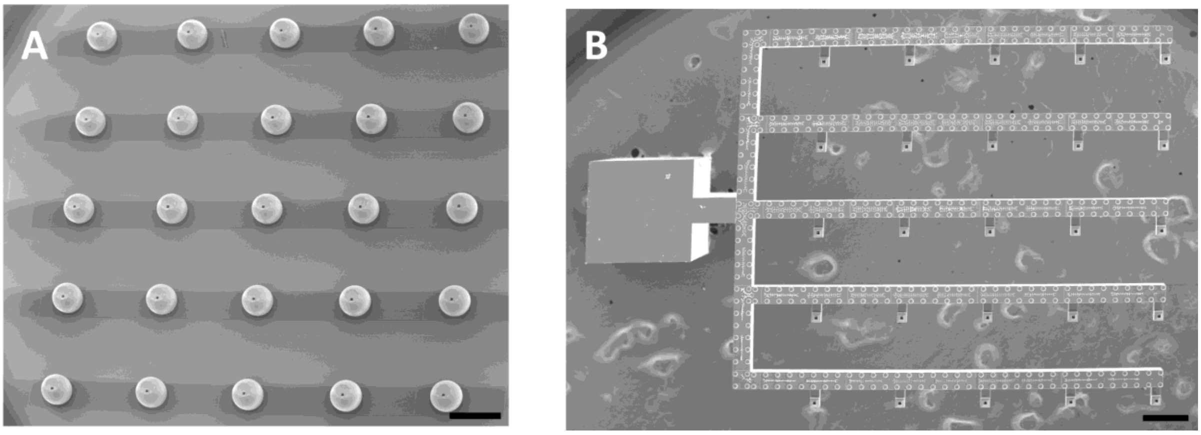

Figure S5. Separation of a microrobot array from microfluidic channels. (**A**) SEM micrograph of the microrobot array after separation from the microfluidic channels. Scale bar: 500 µm. (**B**) SEM micrograph of the microfluidic channels after separation from the microrobot array. Scale bar: 500 µm.

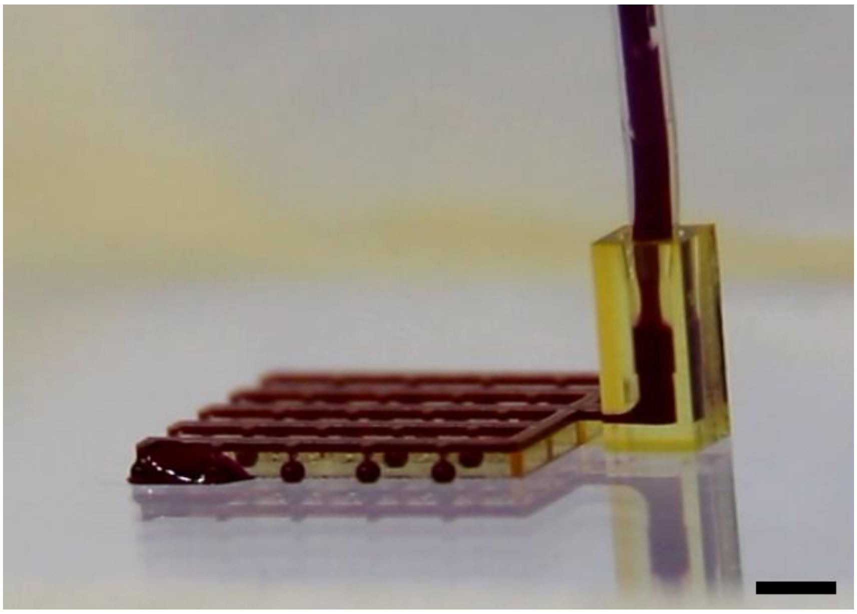

Figure S6. Loading of the microrobot array by the microfluidic platform in the absence of the pressure release chamber. Non-uniform loading and leakage of cargo were both observed. Scale bar: 1 mm.

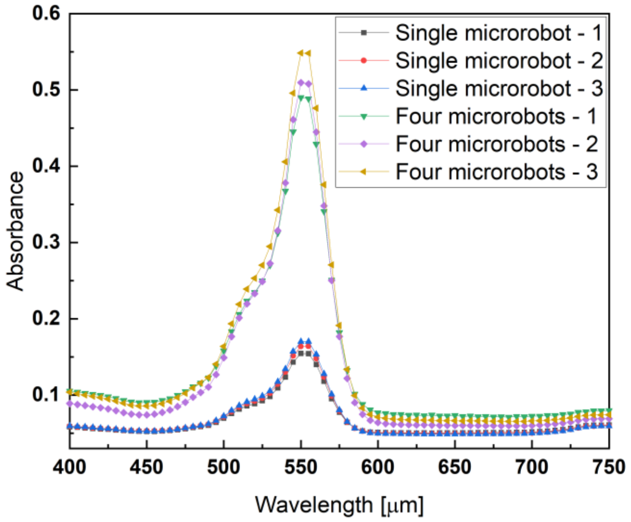

Figure S7. Quantification of the microfluidic loading process across the microrobot array. Two groups each of a single microrobot and four microrobots were placed in 400 µl PBS. UV-VIS spectra of the solutions from each group were compared to evaluate cargo loading.

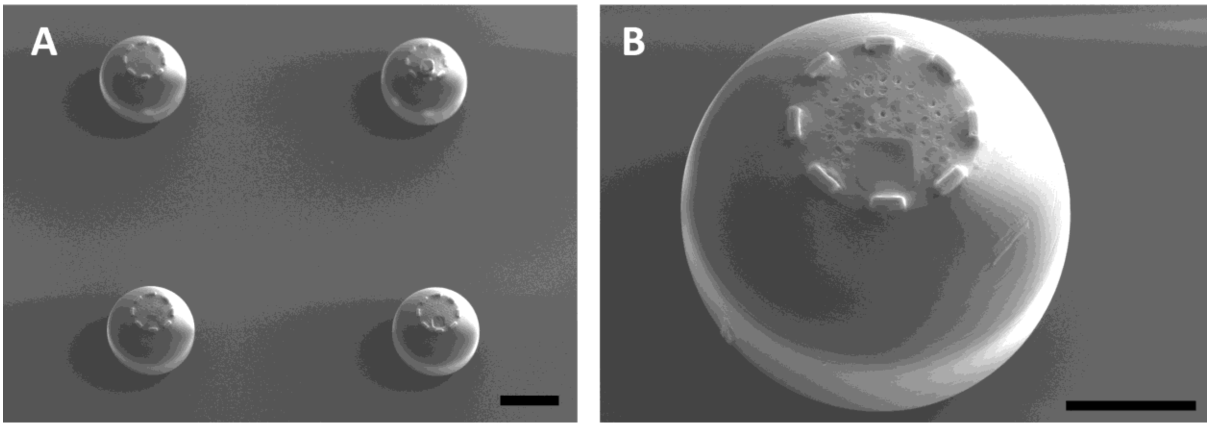

Figure S8. SEM micrographs of PCL-sealed microrobots. (**A**) Sealed microrobot array. Scale bar: 200 µm. (**B**) Close view of a single sealed microrobot. The 45 µm square inlet allows a microfluidic channel to be inserted during loading. Scale bar: 100 µm.

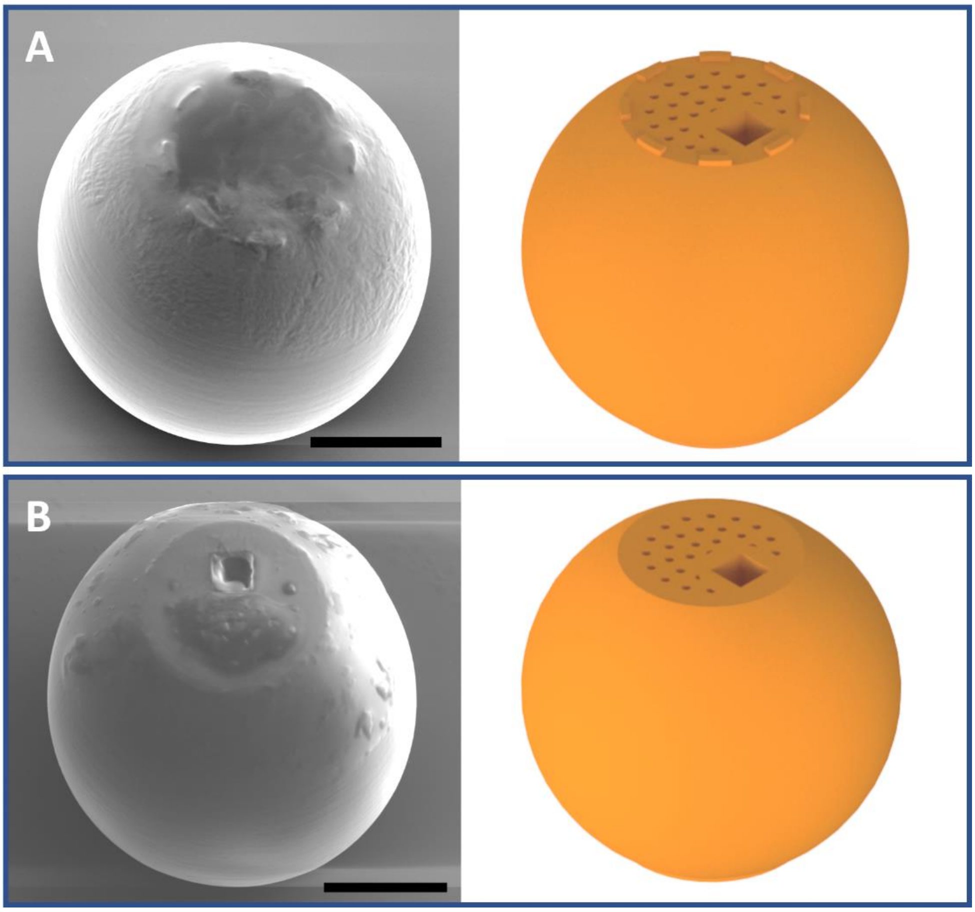

Figure S9. SEM micrographs comparing PCL dip-sealing of microrobots with and without protrusions. (**A**) Sealing in the presence of protrusions. (**B**) Sealing in the absence of protrusions. Visual inspection shows that the dip-sealing process improves in the presence of protrusions. All scale bars: 100 µm.

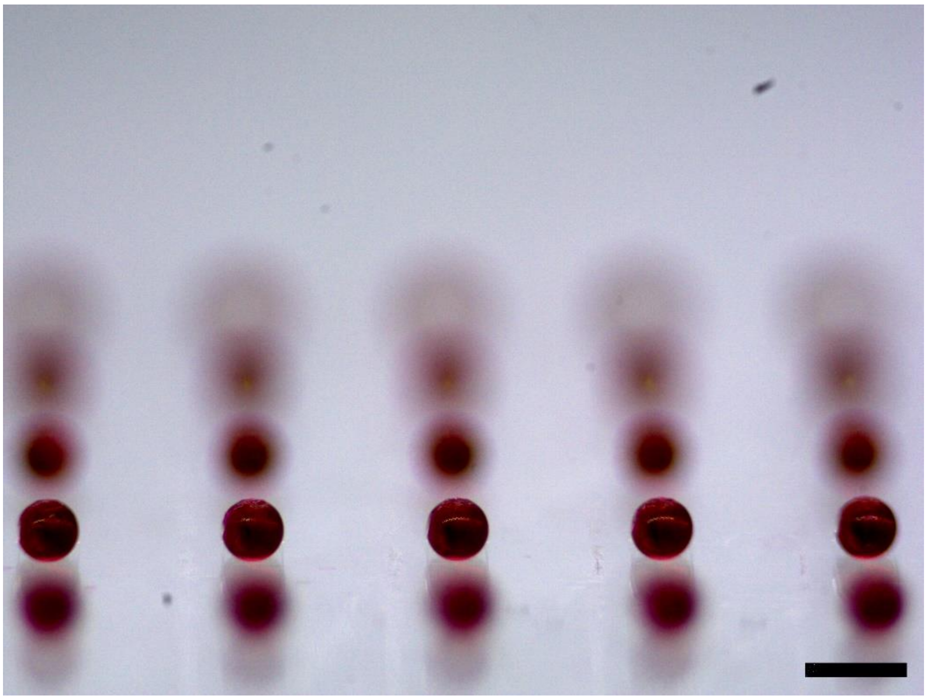

Figure S10. Optical images of NIR-PCL-sealed microrobots. An array of 25 loaded microrobots sitting on the glass substrate. Scale bar: 500 µm.

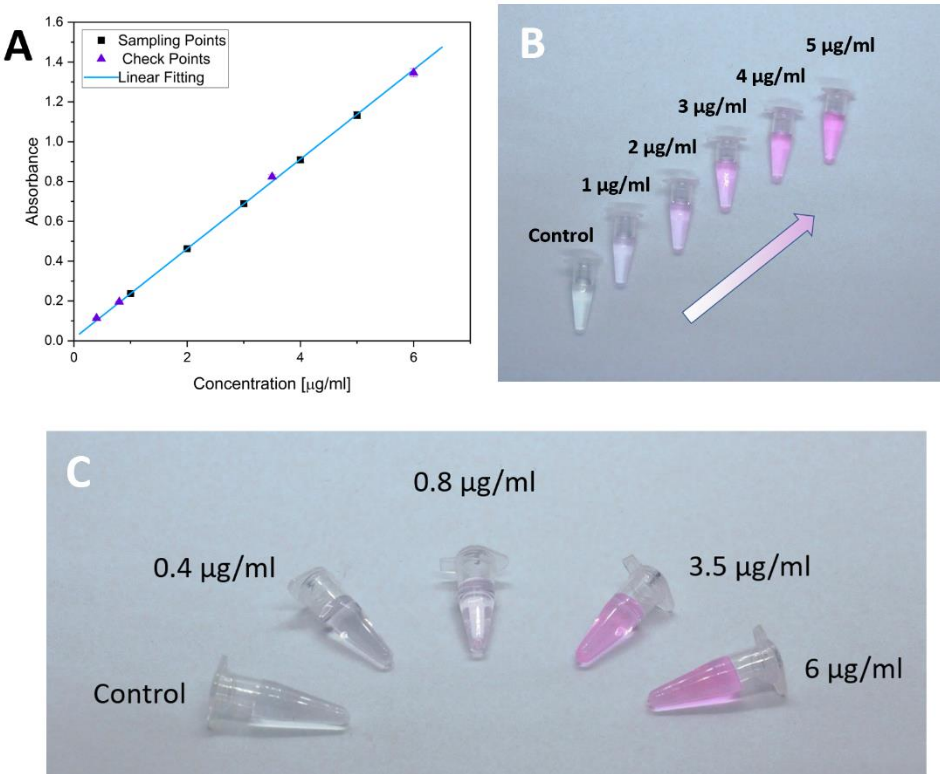

Figure S11. Correlation of rhodamine B concentration and absorbance. (**A**) Rhodamine B absorbance vs concentration dose-response curve. A linear fitting was obtained based on five concentrations: 1 µg·ml^{-1}, 2 µg·ml^{-1}, 3 µg·ml^{-1}, 4 µg·ml^{-1}, and 5 µg·ml^{-1}. Four additional concentrations were employed to validate the curve: 0.4 µg·ml^{-1}, 0.8 µg·ml^{-1}, 3.5 µg·ml^{-1}, and 6 µg·ml^{-1}. Data shown as mean, n = 3. (**B**) The five concentrations used to fit the dose-response curve. (**C**) The four concentrations employed to verify the dose-response curve.

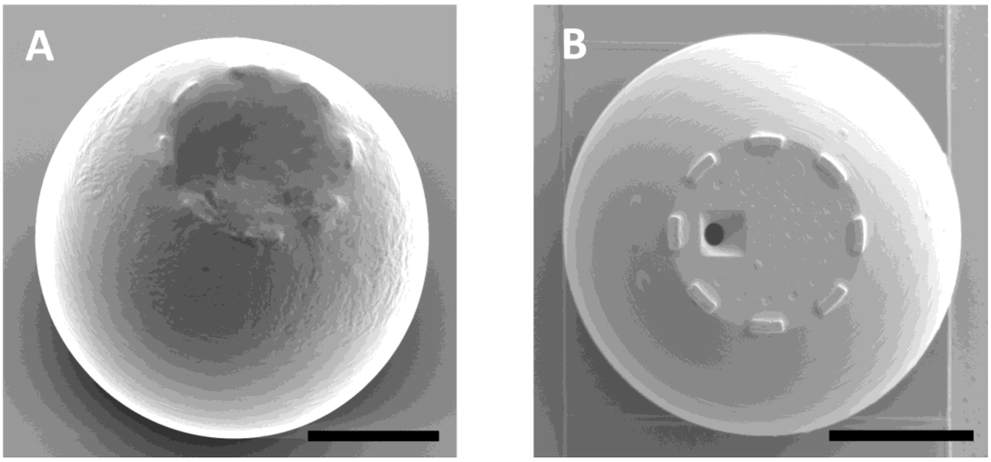

Figure S12. PCL dip-sealed microrobot structure before and after thermal stimulation. SEM micrographs of a fully sealed microrobot (**A**) before and (**B**) after thermally-triggered cargo release. Scale bars: 100 µm.

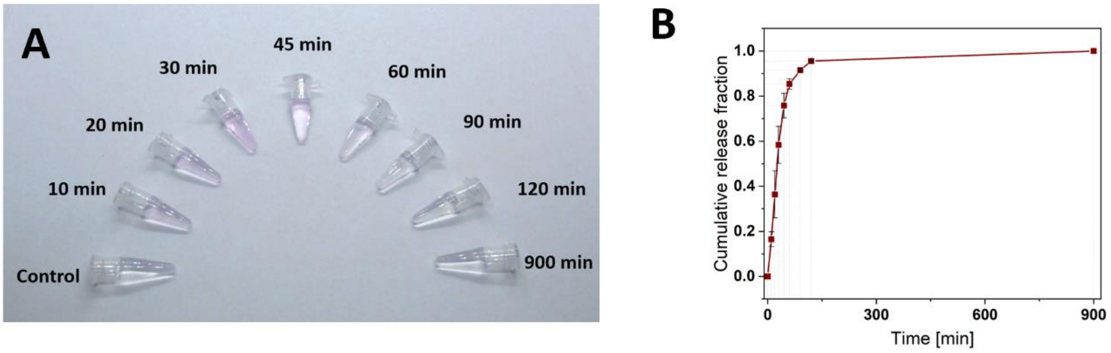

Figure S13. Thermally-triggered cargo release from microrobots at different time points. (**A**) Microrobot incubation solutions at eight time points. (**B**) Cumulative cargo release fraction from microrobots loaded with 2 wt% rhodamine B.

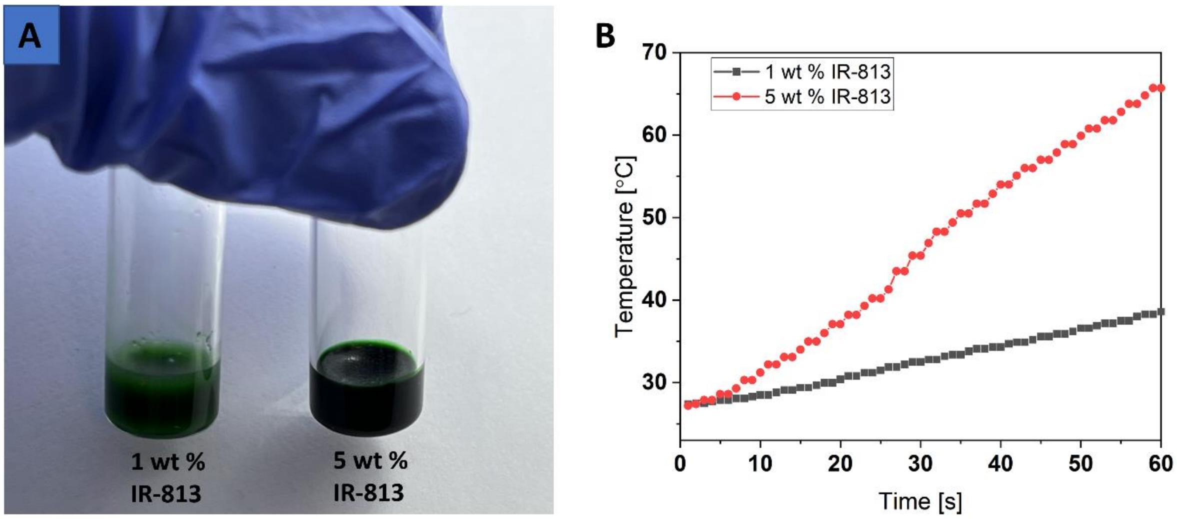

Figure S14. Photothermal properties of 1 wt % IR-813 and 5 wt % IR-813 PCL resin. (**A**) The colour of the two resins. (**B**) The temperature of the two resins within 60 seconds under NIR exposure.

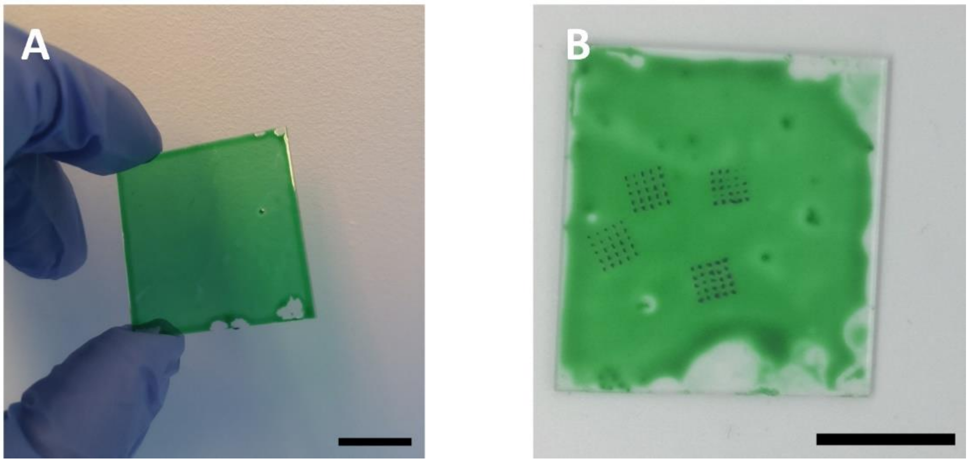

Figure S15. Optical images of the NIR-PCL sealing layer coated on a glass substrate. (**A**) The sealing resin before the dip sealing process. (**B**) The sealing resin after 4 cycles of dip-sealing. Four imprints are clearly visible in the resin. All scale bars: 10 mm.

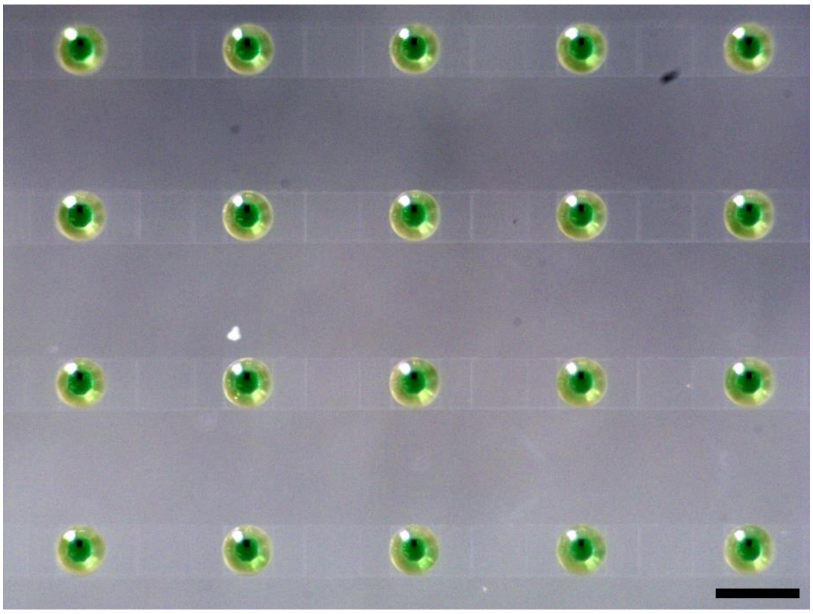

Figure S16. Optical image of an empty microrobot array following dip-sealing with the NIR-PCL resin. Scale bar: 500 μm.

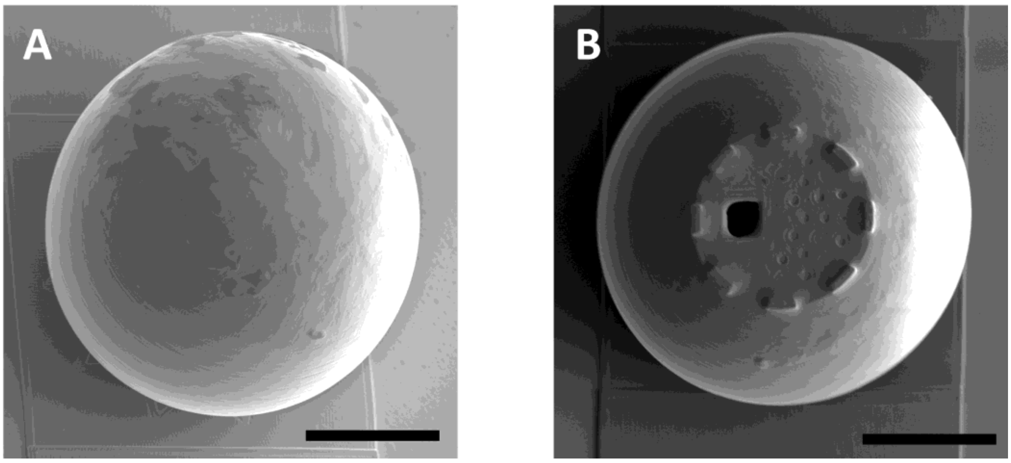

Figure S17. NIR-PCL dip-sealed microrobots before and after NIR irradiation. SEM micrographs of a fully sealed microrobot (**A**) before and (**B**) after NIR-induced cargo release. All scale bars: 100 µm.

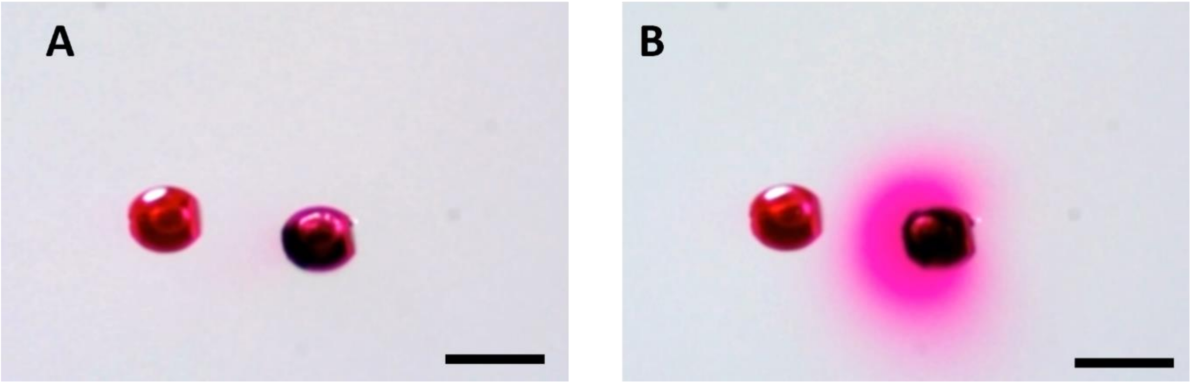

Figure S18. NIR irradiation of microrobots sealed with PCL (left) and NIR-PCL (right). Microrobots (**A**) before and (**B**) after exposure to NIR Microrobots after NIR irradiation. All scale bars: 500 µm.

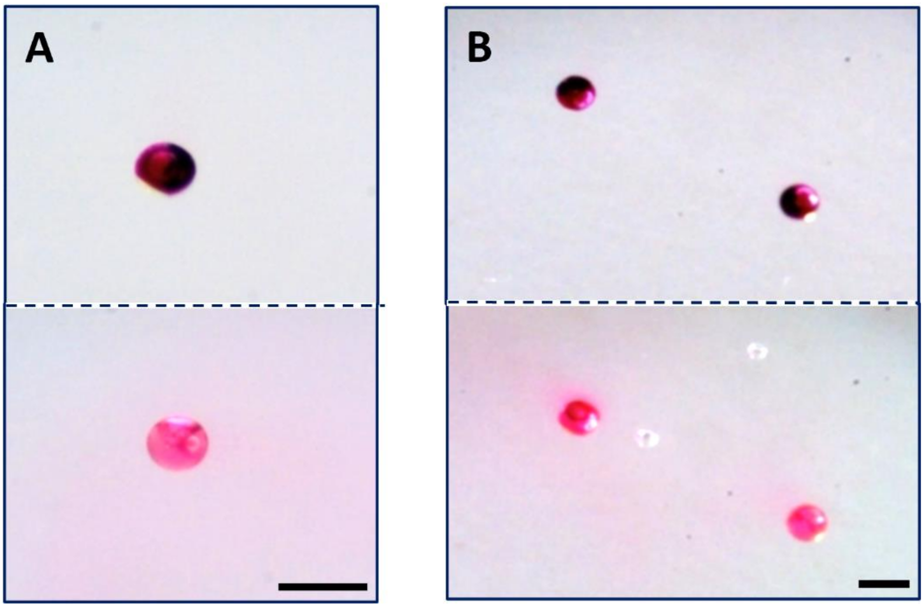

Figure S19. Microrobots during the cargo release process. (**A**) Optical images of a single microrobot before (top) and after (bottom) NIR irradiation. (**B**) Optical images of two microrobots before (top) and after (bottom) NIR irradiation. All scale bars: 500 µm.

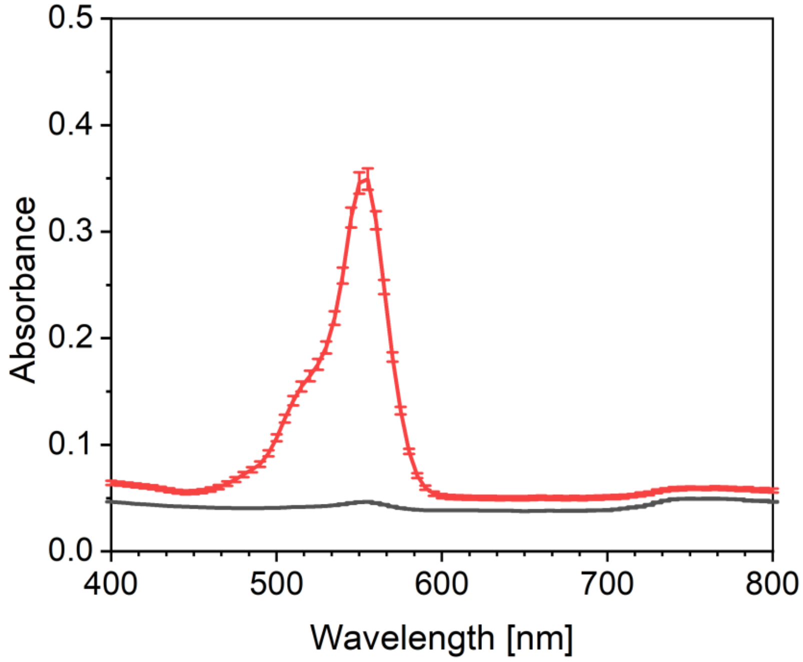

Figure S20. Stability of the NIR-PCL sealed microrobot. UV-VIS spectra of PBS solution containing microrobots following incubation at 4 °C for 3 days. Black and red curves represent sealed and unsealed microrobots, respectively. Data shown as mean ± S.D., n = 3.

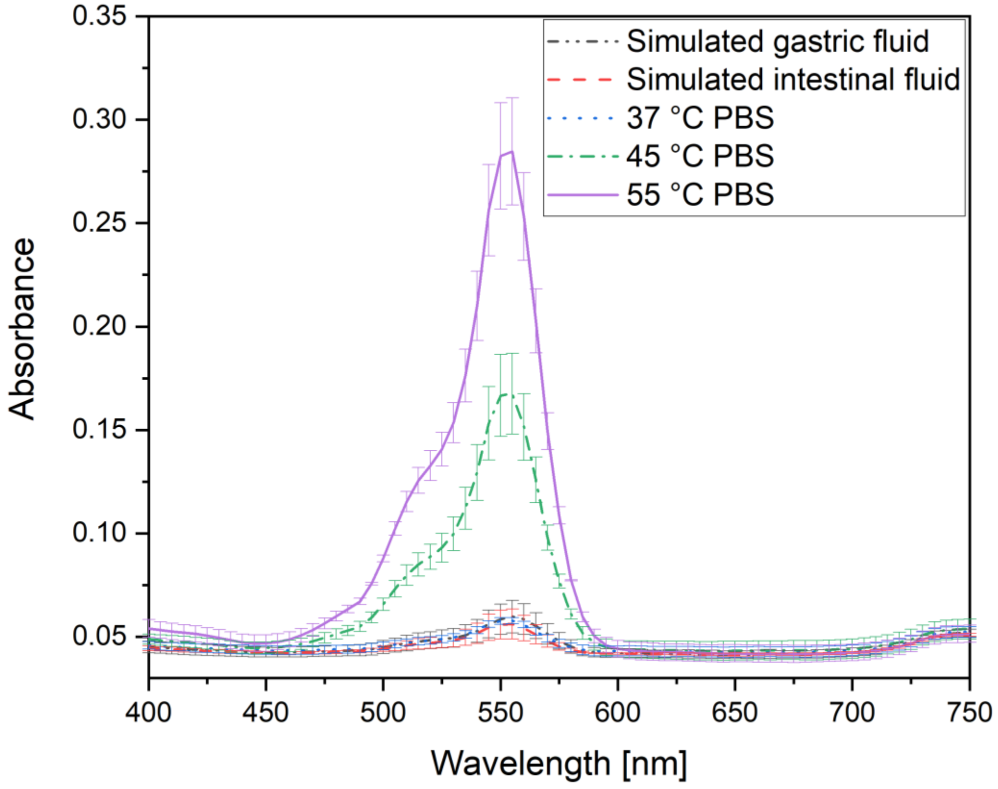

Figure S21. Characterisation of cargo release from the NIR-PCL sealed microrobots under simulated physiological conditions. UV-VIS spectra of the solutions from the sealed microrobots incubated under various conditions: 37 °C simulated gastric fluid, 37 °C simulated intestinal fluid, 37 °C PBS solution, 45 °C PBS solution, and 55 °C PBS solution. Data shown as mean ± S.D., n = 3.

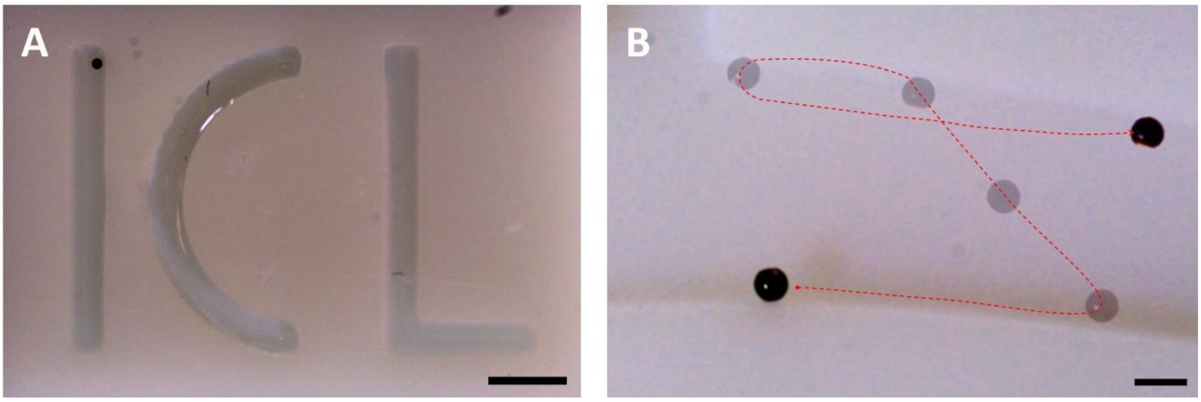

Figure S22. Locomotion of magnetic microrobotic systems. (**A**) The microrobots can freely move inside a constrained channel in the shape of "ICL" (abbreviation of Imperial College London). Scale bar: 2 mm. (**B**) Microrobot motion is flexible and its direction can be changed by varying the magnetic field. Scale bar: 500 µm.

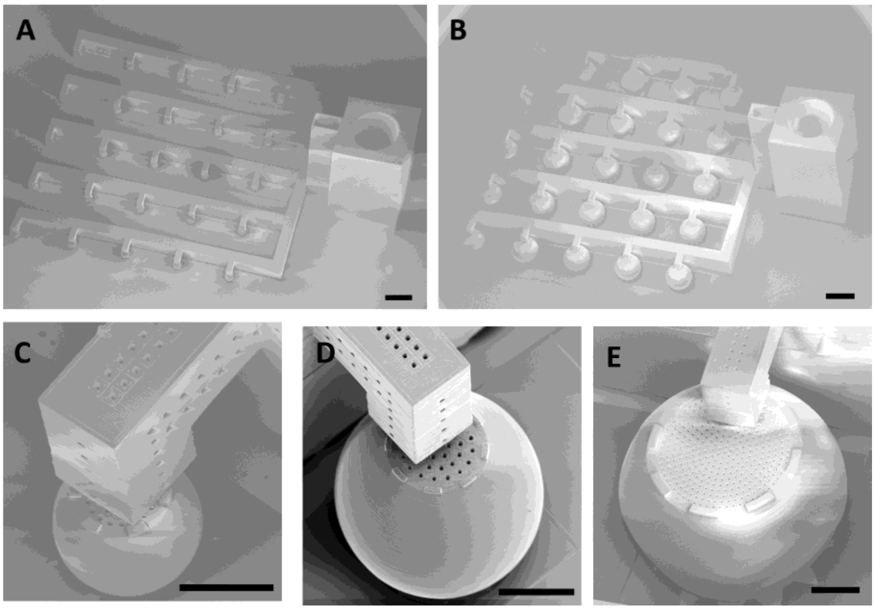

Figure S23. Tailoring the size of microrobots produced by MLDS. SEM micrograph of the overview of the printed microfluidic loading system with (**A**) small microrobots (165 µm) and (**B**) large microrobots (495 µm). Scale bar: 500 µm. SEM micrograph around the interface between the microfluidic channel and microrobot with (**C**) small diameter of 165 µm, (**D**) medium diameter of 310 µm, and (**E**) large diameter of 495 µm. Scale bar: 100µm.

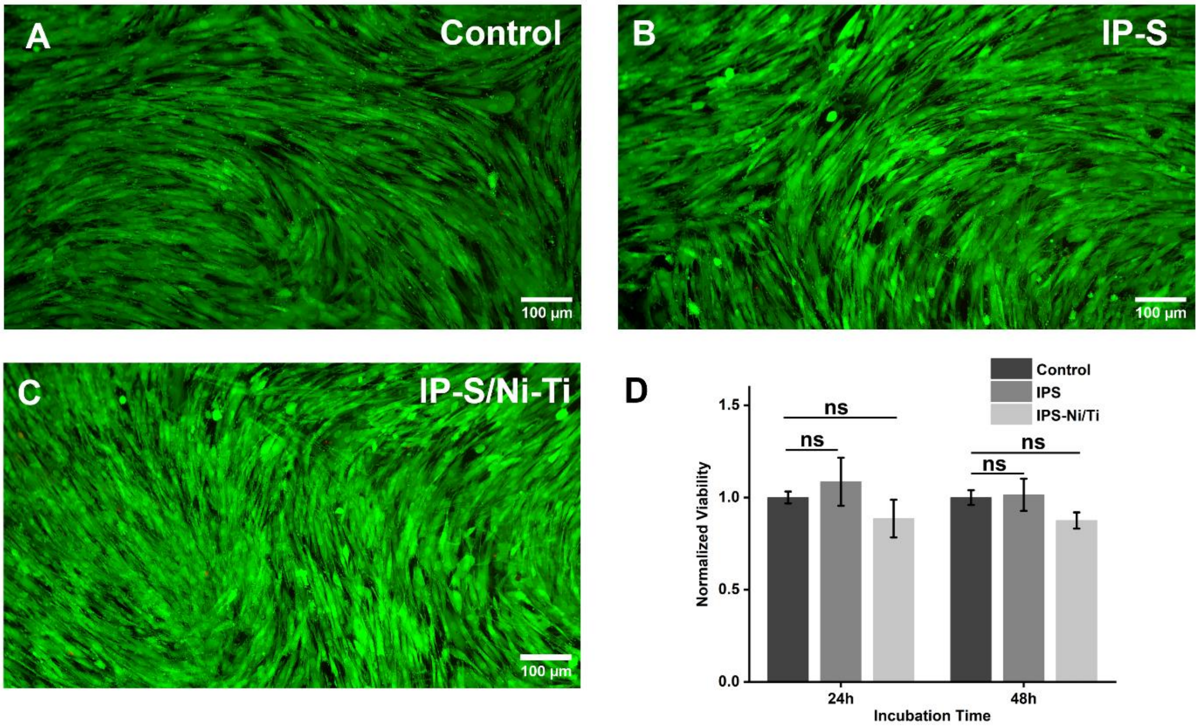

Figure S24. Cell viability in the presence of microrobots. LIVE/DEAD™ staining images of the cell monolayers: (**A**) control, (**B**) incubated with IP-S microrobots, and (**C**) incubated with IP-S microrobots coated with Ni/Ti. (**D**) Cell viability after 24 h and 48 h treatment. The cell viability values are expressed as mean ± SD, $n = 3$. Significance determined by one-way analysis of variance (ANOVA) followed by Dunnett's test, $p = 0.665$ for control vs IP-S (24 h), $p = 0.374$ for control vs IP-S coated with Ni/Ti (24 h), $p = 0.989$ for control vs IP-S (48 h), $p = 0.054$ for control vs IP-S coated with Ni/Ti (48 h).

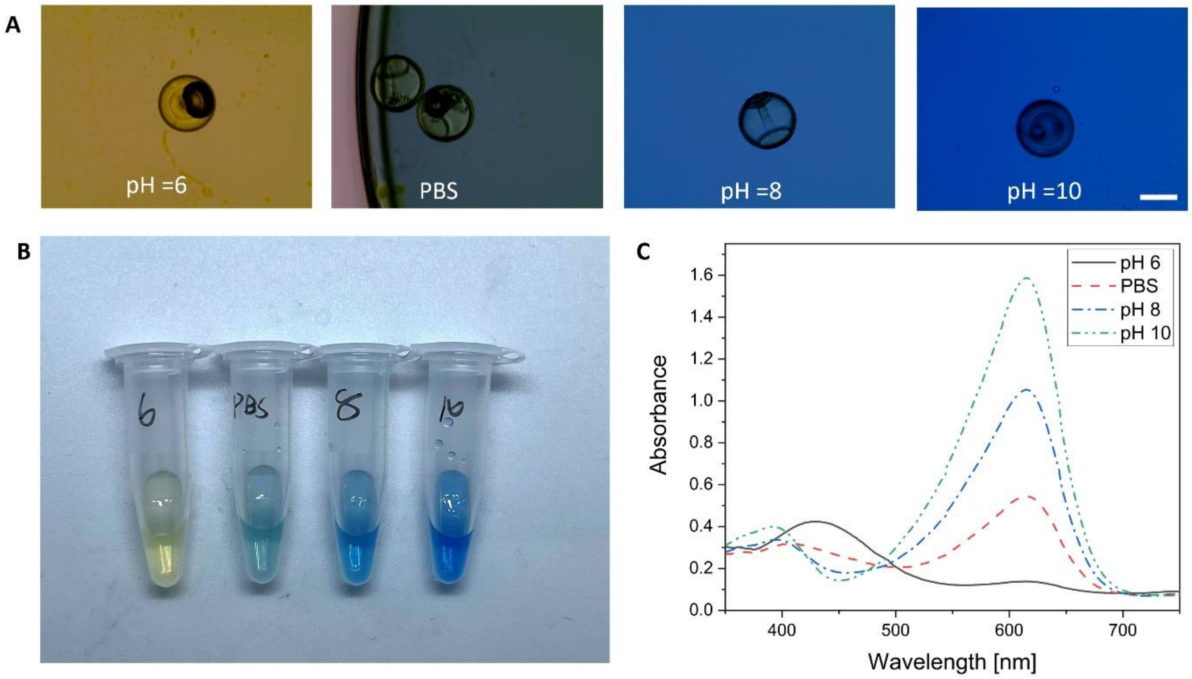

Figure S25. Sensing accuracy of BB-loaded microrobots in different pH solutions. (A) The released microrobots in solutions of pH 6, 8, and 10 solutions and PBS. Scale bar: 200 µm. (B) The solutions showed visible colour differences. (C) UV-VIS spectra of the solutions.

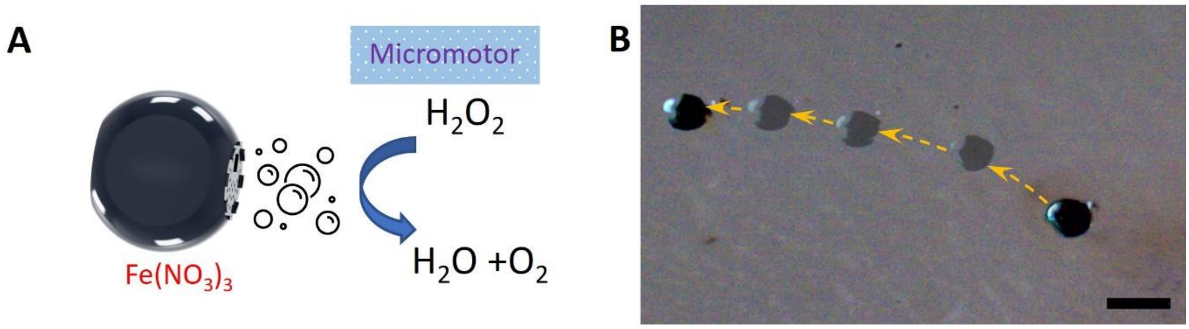

Figure S26. Proof-of-concept of microrobots as micromotors. (**A**) Schematic of the working principle underpinning chemically-powered micromotors. In brief, bubbles are generated by a chemical reaction which is initiated when the encapsulated catalyst solution encounters the surrounding solution (chemical fuel). (**B**) Trajectory of a micromotor powered by bubbles generated around the lid of the microrobot. Scale bar: 500 µm.

Legends of supplementary movies

Movie S1. Schematics of i) the fabrication process of MLDS and ii) its potential applications.

Movie S2. The overall process of microfluidic loading of microrobots with rhodamine B solution.

Movie S3. A close view of the loading process showing cargo flowing from the channel into the chambers of the microrobots.

Movie S4. 3D confocal image of a fully loaded microrobot.

Movie S5. The dip-sealing process.

Movie S6. Cargo release from the sealed microrobots when the temperature of the surrounding environment was above the melting point of PCL sealing resin.

Movie S7. Real-time release of rhodamine B from the microrobots on the glass substrate in response to an increase of the environmental temperature.

Movie S8. Photothermal analysis of the NIR-PCL resin.

Movie S9. NIR irradiation of microrobots sealed with PCL and NIR-PCL.

Movie S10. Selective cargo release from microrobots sealed with NIR-PCL.

Movie S11. Quantification of cargo release from one and two microrobots.

Movie S12. Various locomotion control studies of the magnetic microrobot.

Movie S13. Targeted motion control and NIR-triggered cargo release in a printed vascular-like environment.

Movie S14. MLDS microrobots for environmental sensing applications.

Movie S15. MLDS microrobots for micromotor applications.
